# S-palmitoylation of MAP kinase is essential for fungal virulence

**DOI:** 10.1128/mbio.02704-24

**Published:** 2024-10-29

**Authors:** Yuhang Duan, Pingping Li, Deyao Zhang, Lili Wang, Yuan Fang, Hong Hu, Qiulu Mao, Xiaolan Zhou, Panpan Zhao, Xuechun Li, Jinfeng Wei, Jintian Tang, Li Pan, Hao Liu, Xiaolin Chen, Xiaoyang Chen, Tom Hsiang, Junbin Huang, Lu Zheng

**Affiliations:** 1 State Key Laboratory of Agricultural Microbiology/Hubei Key Laboratory of Plant Pathology, Huazhong Agricultural University, Wuhan, China; 2 Anhui Province Key Laboratory of Crop Integrated Pest Management/College of Plant Protection, Anhui Agricultural University, Hefei, China; 3 Zhejiang Provincial Key Laboratory of Biometrology and Inspection & Quarantine, College of Life Sciences, China Jiliang University, Hangzhou, China; 4 Life Science and Technology Center, China Seed Group Co,. Ltd, Wuhan, China; 5 School of Environmental Sciences, University of Guelph, Guelph, Canada; Duke University Hospital, Durham, North Carolina, USA

**Keywords:** S-palmitoylation, *Ustilaginoidea virens*, virulence

## Abstract

S-palmitoylation is an important reversible protein post-translational modification in organisms. However, its role in fungi is uncertain. Here, we found the treatment of the rice false fungus *Ustilaginoidea virens* with S-palmitoylation inhibitor 2 BP resulted in a significant decrease in fungal virulence. Comprehensive identification of S-palmitoylation sites and proteins in *U. virens* revealed a total of 4,089 S-palmitoylation sites identified among 2,192 proteins and that S-palmitoylated proteins were involved in diverse biological processes. Among the five palmitoyltransferases, UvPfa3 and UvPfa4 were found to regulate the pathogenicity of *U. virens*. We then performed quantitative proteomic analysis of ∆*UvPfa3* and ∆*UvPfa4* mutants. Interestingly, S-palmitoylated proteins were significantly enriched in the mitogen-activated protein kinase and autophagy pathways, and MAP kinase UvSlt2 was confirmed to be an S-palmitoylated protein which was palmitoylated by UvPfa4. Mutations of S-palmitoylation sites in *UvSlt2* resulted in significantly reduced fungal virulence and decreased kinase enzymatic activity and phosphorylation levels. Simulations of molecular dynamics demonstrated mutation of S-palmitoylation sites in *UvSlt2* causing decreased hydrophobic solvent-accessible surface area, thereby weakening the bonding force with its substrate UvRlm1. Taken together, S-palmitoylation promotes *U. virens* virulence through palmitoylation of MAP kinase UvSlt2 by palmitoyltransferase UvPfa4. This enhances the enzymatic phosphorylation activity of the kinase, thereby increasing hydrophobic solvent-accessible surface area and binding activity between the UvSlt2 enzyme and its substrate UvRlm1. Our studies provide a framework for dissecting the biological functions of S-palmitoylation and reveal an important role for S-palmitoylation in regulating the virulence of the pathogen.

**IMPORTANCE:**

S-palmitoylation is an important post-translational lipid modification of proteins. However, its role in fungi is uncertain. In this study, we found that S-palmitoylation promotes virulence of rice false smut fungus *U. virens* through palmitoylation of MAP kinase UvSlt2 by palmitoyltransferase UvPfa4. This enhances the enzymatic phosphorylation activity of the kinase, thereby increasing hydrophobic solvent-accessible surface area and binding activity between the UvSlt2 enzyme and its substrate UvRlm1. Our studies provide a framework for dissecting the biological functions of S-palmitoylation and reveal an important role for S-palmitoylation in regulating the virulence of the pathogen. This is the first functional study to reveal the role of S-palmitoylation in fungal virulence.

## INTRODUCTION

Post-translational modification regulates numerous biological processes including growth, development, and pathogen virulence. Many types of post-translational modifications, including phosphorylation, methylation, acetylation, and ubiquitination, regulate gene expression in eukaryotes and play different roles in protein structure, stability, activity, localization, and interaction ability. Palmitoylation, the post-translational modification of proteins with the lipid palmitate, has emerged as an important mechanism for regulating protein localization, secretion, stability, and function by altering membrane affinity. S-palmitoylation is an important form of lipidation modification (1), which promotes the trafficking of proteins to lipid rafts and membrane microdomains rich in cholesterol and sphingolipids (2). Unlike other lipid modifications, this lipid modification is reversible and regulates protein function in a manner similar to protein phosphorylation or ubiquitination (3). Protein S-palmitoylation is catalyzed by S-acyltransferases (also called palmitoyl acyltransferases, PATs) and is a common mechanism to enhance the overall hydrophobicity of a protein (4). The first palmitic acyltransferase, Erf2p/Erf4p complex, was discovered in yeast. Ras palmitoyltransferase activity was abolished by mutating conserved residues in the DHHC-CRD domain of Erf2P (C189, H201, and C203), which eliminated its palmitoyl transferase activity (5). Previous studies indicated that palmitoylation can participate in small GTPase signaling, calcium signaling, heterotrimeric G-protein signaling, and disease resistance in plants. The *Arabidopsis* gene PAT24 is involved in the proliferation of both tip and diffuse cells (6). PAT4 may have a similar function by fine-tuning the activities of Rho-related proteins in plants (7). PAT10 mutant plants have deficiencies in development, vascular patterning, fertility, and salt tolerance (8). PAT15 is involved in β-oxidation of triacylglycerol during early seedling growth, and PAT21 is required for repairing DNA double-strand breaks (9, 10). However, the function of S-palmitoylation in the cellular processes of filamentous fungi remains unknown, thus hindering the understanding of the broad biological functions of cysteine palmitoylation in fungi.

Hundreds of mammalian proteins have been identified to be palmitoylated, many of which are associated with cancer, inflammation, and neurological diseases, among others (11). Palmitoylation of PD-L1 significantly inhibits PD-L1 ubiquitination, thereby preventing PD-L1 degradation and inhibiting tumor killing by T cells (12). NOD1 and NOD2 are correctly localized to the plasma membrane after palmitoylation and can recognize and induce NOD1/2-mediated immune responses in cells (13). In *Arabidopsis*, S-acylation of NB-LRR protein R5L1 by palmitic acyltransferase PAT13 and PAT16 mediates the plant disease resistance reaction (14). S-palmitoylation of *Arabidopsis* palmitic acyltransferase PAT5 and PAT9 controls the activity of immune receptor P2K1 by antagonizing protein phosphorylation and protein degradation, and further negatively regulates eATP-induced innate immune signal transduction in plants (15). S-palmitoylation has also been reported in biological functions of yeast, where S-palmitoylation of Tlg1 by palmitic acyltransferase Swf1 maintains protein stability, thereby preventing its ubiquitination and degradation (16). S-palmitoylation of yeast vacuolar protein Vac8 by palmitic acyltransferase Pfa3 is important for its localization, vacuolar morphology, and inheritance (17). Palmitoylation by the DHHC protein Pfa4 regulates the ER exit of Chs3 (18). The palmitic acyltransferase Akr1 is capable of S-acylation of the Yck2 protein kinase, targeting Yck2 to the plasma membrane (19). However, the pathogenic mechanism of S-palmitoylation in fungi has not been reported, thus hindering the understanding of the broad biological functions of S-palmitoylation in plant pathogenic fungi.

Rice false smut, caused by the pathogenic ascomycetous fungus *Ustilaginoidea virens*, is one of the most devastating grain diseases in rice-growing areas of the world (20). The occurrence of this disease not only leads to a reduction in crop yield but also poses a threat to human or animal health through the production of cyclopeptide mycotoxins (20–26). S-palmitoylation, a post-translational modification, could provide a potential new avenue to design strategies aimed at managing rice false smut disease. In this study, treatment of *U. virens* hyphae with palmitoylation inhibitors was found to inhibit the virulence of *U. virens*. We then used a proteomics approach to quantify S-palmitoylation sites of *U. virens*. We also revealed that S-palmitoylation plays key roles in multiple conserved cellular processes, fungal development, and virulence of *U. virens*. S-palmitoylation of *U. virens* MAP kinase UvSlt2 by the palmitoyltransferase UvPfa4 promoted protein phosphorylation activity and thus enhanced the virulence of the pathogen. This is the first functional study to reveal the role of S-palmitoylation in fungal virulence.

## RESULTS

### S-palmitoylation regulates the development and virulence of *U. virens*

**Fig 1:**
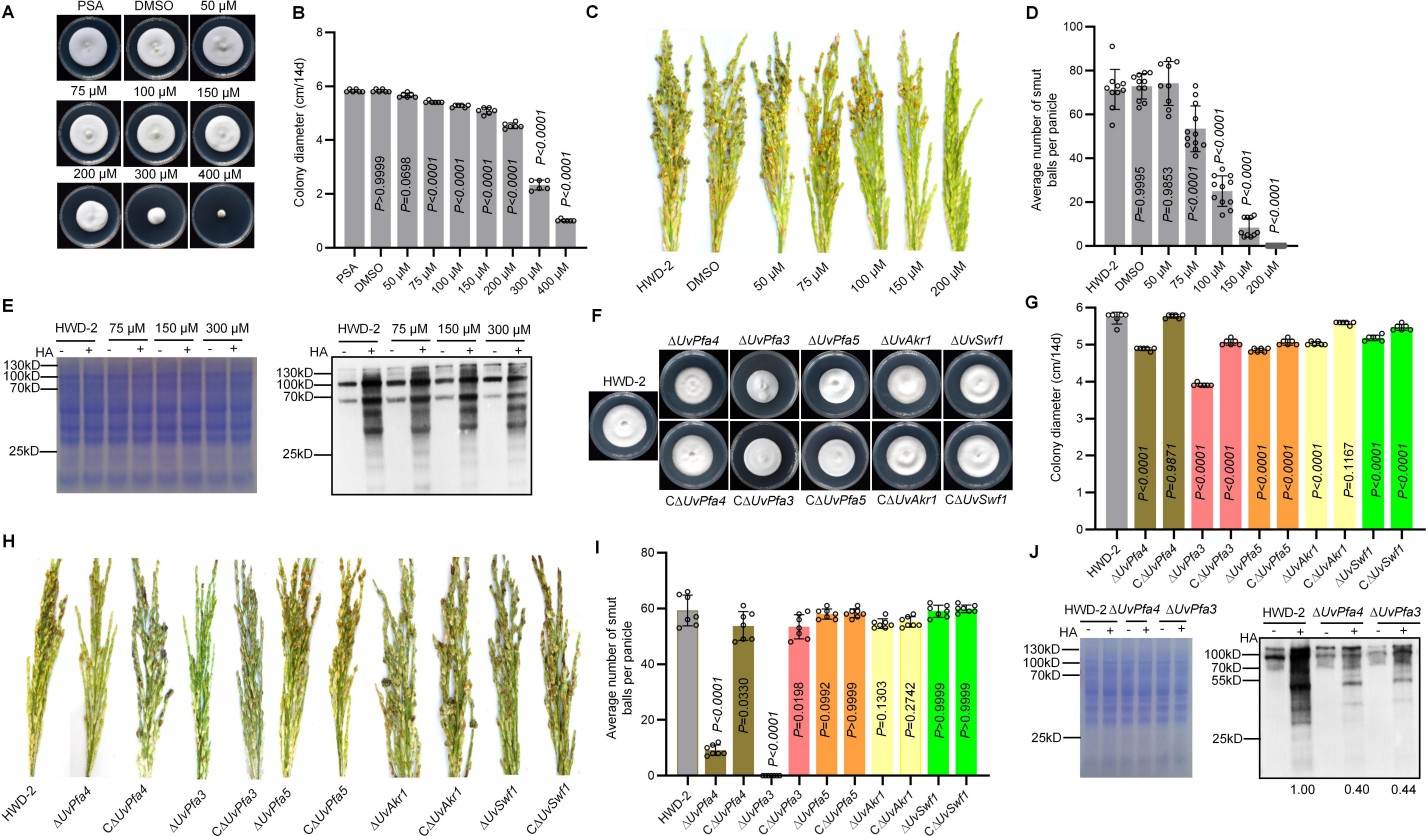
S-palmitoylation regulates the development and virulence of *U. virens*. (**A**) Colonies of the *U. virens* wild-type strain HWD-2 on PSA medium containing different concentrations of 2 BP after incubation for 14 d at 28°C. (**B**) Colony diameters of wild-type HWD-2 on PSA medium containing different concentrations of 2 BP after 14 d at 28°C. (**C**) Virulence assays of the wild-type HWD-2 treated with different concentrations of 2 BP on rice spikelets at 21 dpi. (**D**) The mean number of rice smut balls per panicle. Data were collected from three independent experiments for each treatment. Nine rice panicles were inoculated per replicate. (**E**) Western blot analysis of palmitoylated proteins in *U. virens* after treatment with 2 BP by the acyl-biotin exchange (ABE) method. Loading quantity was determined by coomassie bright blue (CBB). HA: hydroxylamine. +: total protein treated with hydroxylamine. −: total protein not treated with hydroxylamine. Values represent the relative signal intensity of each band, and the HWD-2 sample was set to 1.00. (**F**) Palmitoyltransferase family mutant and wild-type strains on PSA for 14 days at 28°C. (**G**) Colony diameters of the palmitoyltransferase family mutants on PSA after 14 d. (**H**) Virulence assays of *U. virens* palmitoyltransferase family mutants on rice spikelets at 21 dpi. (**I**) The mean number of rice smut balls per panicle. Data were collected from three independent experiments for each treatment. Seven rice panicles were inoculated per replicate. (**J**) Western blot analysis of palmitoylated proteins in *U. virens* palmitoyltransferase family mutants by the acyl-biotin exchange (ABE) method. Loading quantity was determined by Coomassie bright blue (CBB). HA: hydroxylamine. +: total protein treated with hydroxylamine. −: total protein not treated with hydroxylamine. Values represent the relative signal intensity of each band, and the HWD-2 sample was set to 1.00.

The irreversible S-palmitoylation inhibitor 2-bromopalmitate (2 BP) was first used to determine whether S-palmitoylation could affect the development and virulence of *U. virens*. We tested *U. virens* growth on potato sugar agar (PSA) medium with different concentrations of 2 BP and found a significant reduction in the mycelial growth rate of *U. virens* at high concentrations of 2 BP (Fig. 1A and B). We then measured the conidiation of *U. virens* on potato sugar broth (PSB) medium containing 2 BP. The conidiation was significantly reduced with lower concentrations of 2 BP (Fig. S1A). In pathogenicity tests, treatment of *U. virens* hyphae with different concentrations of 2 BP resulted in reduced or even complete loss of the virulence of *U. virens* (Fig. 1C and D). Western blotting revealed that S-palmitoylation modification in mycelia of *U. virens* gradually decreased with the increase of 2 BP inhibitor concentration (Fig. 1E). These results suggested that S-palmitoylation might regulate the mycelial growth, conidiation, and virulence of *U. virens*.

To further reveal the role of palmitoylation modification in *U. virens,* we identified six palmitoyltransferases in the *U. virens* genome based on the presence of the conserved DHHC-CRD catalytic domain. Phylogenetic analysis of DHHC-CRD catalytic domain protein homologs from different fungi revealed that these proteins are conserved among filamentous fungi (Fig. S2A). Protein domain analysis showed that all palmitoyltransferases in *U. virens* contained both the DHHC-CRD catalytic domain and transmembrane domain (TM) (Fig. S2B). We then examined the expression profiles of these six palmitoyltransferase genes in *U. virens* at various stages of infection by RT-qPCR. The results showed that palmitoyltransferase family proteins were highly expressed at different infection stages, indicating that they played various roles in infection (Fig. S3). To investigate whether palmitoyltransferase genes are associated with the development and virulence of *U. virens*, we generated five palmitoyl transferase gene knockout mutants using a homologous recombination strategy, which was confirmed by multiple PCR analysis (Fig. S4).

Compared to that of HWD-2, the mycelial growth rates of the ∆*UvPfa3,* ∆*UvPfa4,* ∆*UvPfa5,* ∆*UvAkr1,* and ∆*UvSwf1* mutants were significantly reduced (Fig. 1F and G). After mycelia were stained with CFW, we found that the length of the hyphal tip cells was significantly shortened in Δ*UvPfa3* and Δ*UvPfa4* mutants (Fig. S5). We also assessed conidiation of the *U. virens* mutants, Compared to wild-type HWD-2, and while conidial production was significantly increased in the ∆*UvPfa4* and ∆*UvSwf1* mutants, it was significantly reduced in the ∆*UvPfa3,* ∆*UvPfa5,* and ∆*UvAkr1* mutants (Fig. S1B**,** Supporting Information). These results suggested that palmitoyltransferase genes are required for mycelial growth and conidiation of *U. virens*.

Since palmitoyltransferase genes regulated the development of *U. virens*, we further clarified whether palmitoyltransferases are involved in the regulation of *U. virens* response to different stresses. These results indicated that palmitoyltransferase genes can regulate the responses of *U. virens* to osmotic stress and oxidative stress, as well as cell wall integrity (Fig. S6).

We further conducted virulence assays of the palmitoyltransferase mutants on the susceptible rice cultivar Wanxian-98. Virulence was significantly reduced in the ∆*UvPfa4* mutant but completely lost in the ∆*UvPfa3* mutant compared to the wild type (Fig. 1H and I). We then observed the infection processes of ∆*UvPfa3* in rice spikelets (Fig. S7A) and found that hyphae of the ∆*UvPfa3* mutant did not colonize the surfaces of rice filaments at 6 dpi. These results suggested that ∆*UvPfa3* and ∆*UvPfa4* positively regulate the virulence of *U. virens*.

To determine the role of palmityltransferase genes on S-palmitoylation modification of proteins in *U. virens,* the acyl-biotin exchange (ABE) method was used to test the change of S-palmitoylation modification levels in the ∆*UvPfa3* and ∆*UvPfa4* mutants. The results showed that S-palmitoylation modification was significantly attenuated in ∆*UvPfa3* and ∆*UvPfa4* mutants compared to the wild type, suggesting that S-palmitoylation plays an important role in the virulence of *U. virens* through the action of palmitoyltransferase (Fig. 1J).

### Identification of S-palmitoylation sites and proteins in *U. virens*

**Fig 2:**
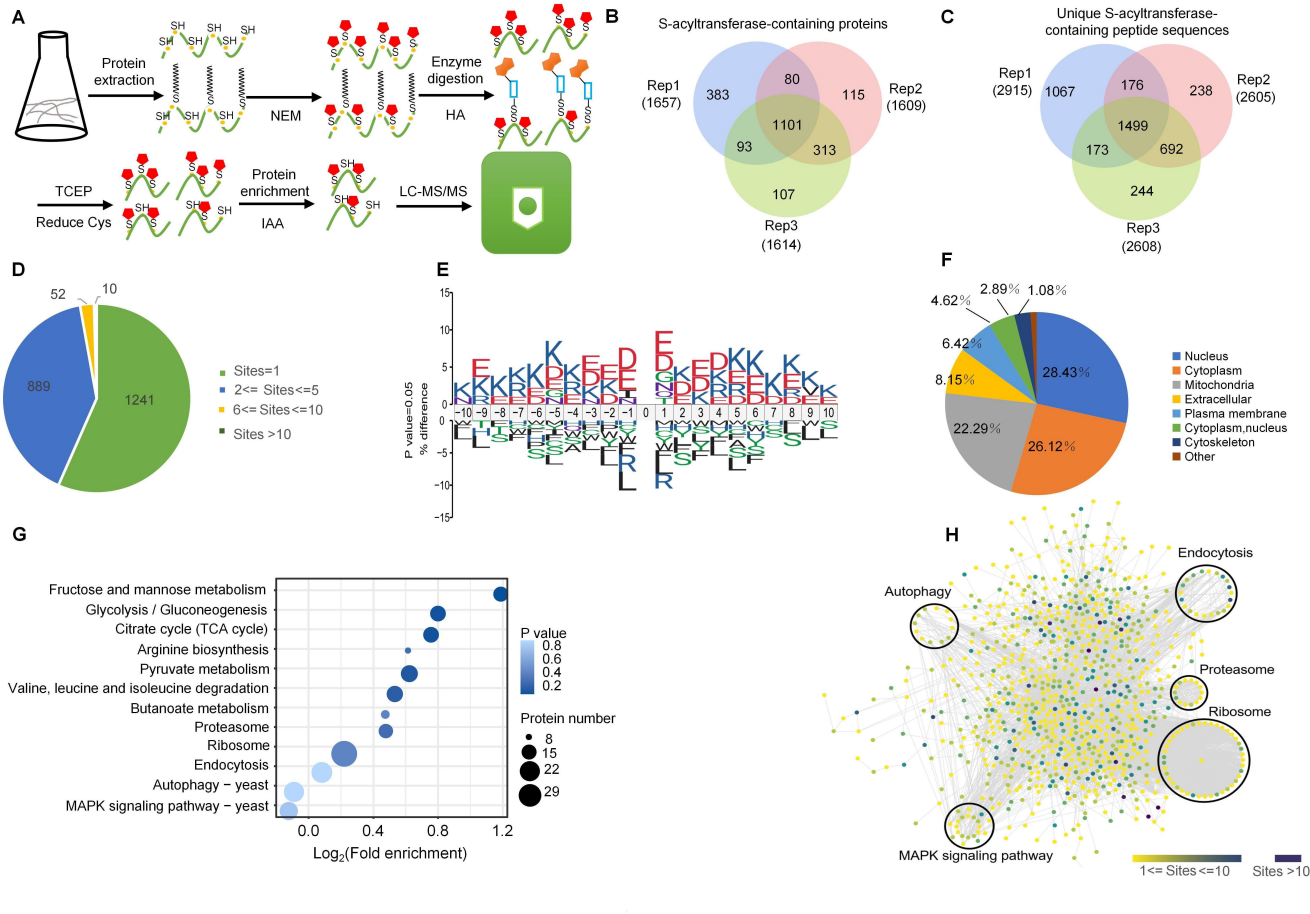
Identification of S-palmitoylation sites and proteins in *U. virens*. (**A**) Identification protocol of proteomics analysis of S-palmitoylation. R-SH, reduced thiols; NEM, N-ethylmaleimide; TCEP, Tris(2-carboxyethyl)phosphine; IAA, Iodoacetamide; HA, hydroxylamine. (**B**) Overlap of S-palmitoylated proteins identified in three biological replicates. (**C**) Overlap of S-palmitoylation sites identified in three biological replicates. (**D**) Distribution of S-palmitoylated proteins based on the number of modification sites. (**E**) Motif analysis of conserved amino acids flanking S-palmitoylation sites. The size of an amino acid reflects the difference in the frequency of an amino acid in the experiment and its frequency in the reference set. The *P* value of each amino acid at every position was calculated by testing the experimental frequency against the frequency of each amino acid in the reference set with Fisher’s Exact test. In this ice Logo, only significant amino acids (*P* < 0.05) are shown. (**F**) Subcellular localization predictions of the S-palmitoylated proteins. (**G**) Kyoto Encyclopedia of Genes and Genomes (KEGG) pathway enrichment analysis of S-palmitoylated proteins. All pathways with *P*-value < 0.05 are shown. (**H**) Protein–protein interaction (PPI) map of S-palmitoylated proteins identified in this study. Proteins in clusters are indicated by red circles.

To identify protein substrates of palmitoyltransferases, we performed a proteomic screen using an affinity-directed MS approach. After LC-MS/MS analyses and database searches (Fig. 2A), the mass errors for S-palmitoylation-containing peptides were <5 ppm, supporting the accuracy of our MS data (Fig. S8A). Most identified peptides varied from 6 to 30 amino acids in length (Fig. S8B). In total, 6,239 S-palmitoylation peptides encompassing 4,089 S-palmitoylation sites from 2,192 proteins were identified at a false discovery rate (FDR) < 1% (Fig. 2B and C; Table S1) from three independent repeat experiments. To examine the distribution of S-palmitoylation sites in individual proteins, we calculated the number of these sites per protein. Whereas 1,241 proteins had only one modification site, 889 proteins had two to five modification sites, 52 proteins had 6 to 10 modification sites, and 10 proteins harbored 10 modification sites (Fig. 2D). Lysine (K) residues were enriched from positions −10, –8 to −4 and +2, +5 to +10 (*P* < 0.05), glutamic acid (E) was over-represented at positions −9, –3, −2 and +1, +3 (*P* < 0.05), and aspartic acid (D) was enriched at the −1 to +4 positions (*P* < 0.05) (Fig. 2E), suggesting that S-palmitoylated proteins shared a common sequence motif.

We further analyzed the subcellular localizations of S-palmitoylated proteins in *U. virens*. Among all 2,192 S-palmitoylation-modified proteins, most were localized to the nucleus (28.43%), cytoplasm (26.12%), or mitochondria (22.29%) (Fig. 2F; Table S2). To better understand the potential functions of S-palmitoylation in *U. virens*, the KEGG pathway enrichment analysis of the S-palmitoylated proteins was conducted. Fructose and mannose metabolism, glycolysis/gluconeogenesis, ribosome, proteasome, endocytosis, autophagy, MAPK signaling pathway, and pyruvate metabolism, as well as valine, leucine, and isoleucine degradation, were found to be significantly enriched (Fig. 2G; Table S4). To explore associations among S-palmitoylated proteins in *U. virens*, we generated a protein interaction network of all these proteins using the STRING database. Some virulence and stress response-related S-palmitoylated proteins were present in the network, including autophagy-related proteins, proteasome, endocytosis pathway proteins, autophagy-related proteins, ribosome structural constituents, and MAPK pathway proteins (Fig. 2H; Table S9). These results reveal candidate proteins for further functional studies aimed at determining the potential roles of S-palmitoylation in important biological processes in *U. virens*.

### Quantitative proteomic analysis of S-palmitoyltransferases ∆*UvPfa3* and ∆*UvPfa4* mutants in *U. virens*

**Fig 3:**
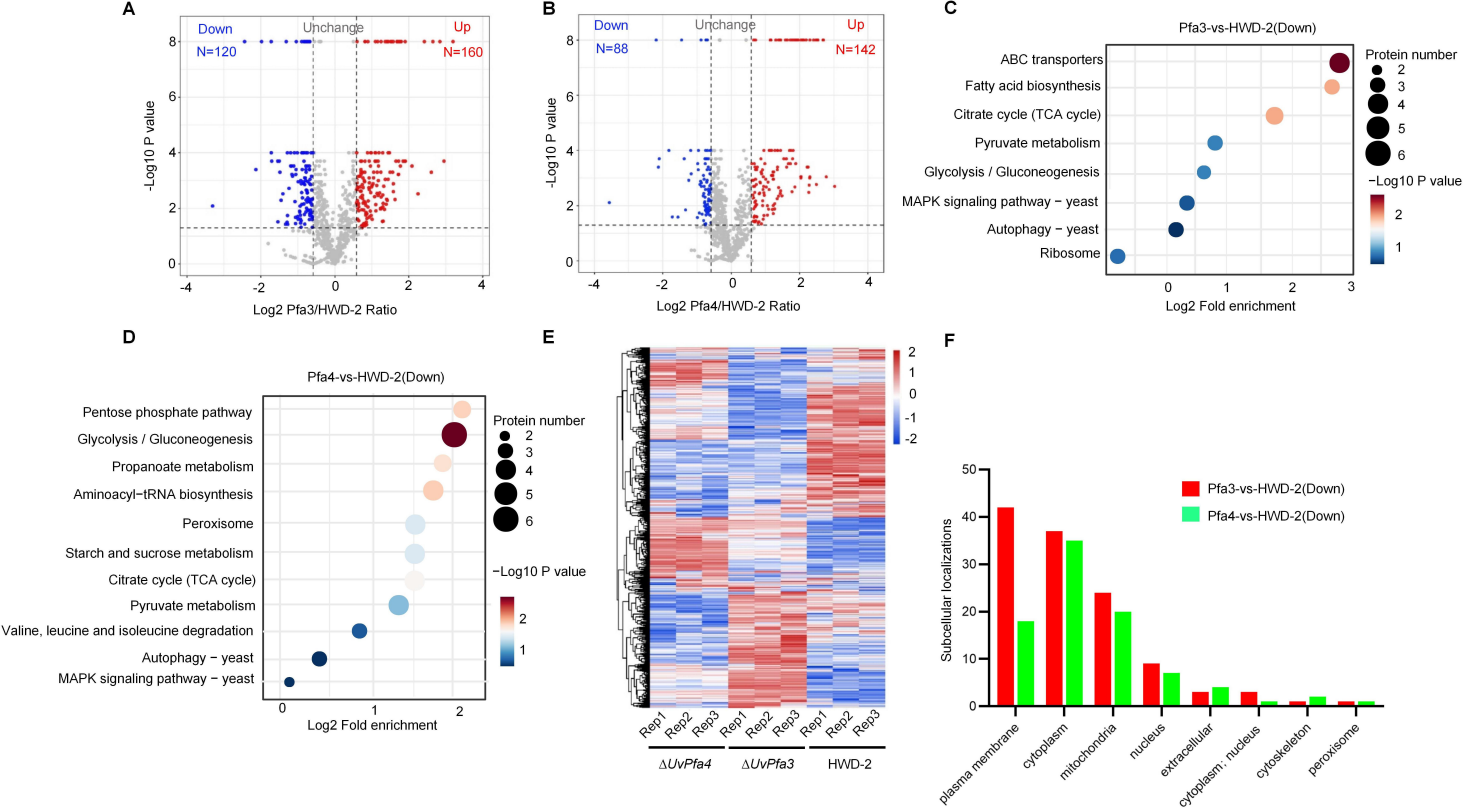
Quantitative proteomic analysis of S-palmitoyltransferases ∆*UvPfa3* and ∆*UvPfa4* mutants in *U. virens*. (**A**) Volcano plots of DEGs from comparative proteomic analysis between ∆*UvPfa3* mutant and HWD-2 strain. HWD-2 strain was used as a control in the comparison group. (**B**) Volcano plots of DEGs from comparative proteomic analysis between ∆*UvPfa4* mutant and HWD-2 strain. HWD-2 strain was used as a control in the comparison group. (**C**) Kyoto Encyclopedia of Genes and Genomes (KEGG) pathway enrichment analysis of downregulated S-palmitoylation proteins in ∆*UvPfa3* mutant. All pathways with *P*-value < 0.05 are shown. (**D**) Kyoto Encyclopedia of Genes and Genomes (KEGG) pathway enrichment analysis of downregulated S-palmitoylation proteins in ∆*UvPfa4* mutant. All pathways with *P*-value < 0.05 are shown. (**E**) Heatmap of differentially-expressed S-palmitoylation proteins in HWD-2, ∆UvPfa3, and ∆Uvpfa4 mutants. (**F**) Subcellular localization of downregulated S-palmitoylation proteins from ∆*UvPfa3* and ∆*UvPfa4* treatments.

To further investigate the pathogenic mechanisms of S-palmitoyltransferases Uvpfa3 and Uvpfa4 in *U. virens*, we compared the S-palmitoylation proteomics of ∆*UvPfa3* and ∆*UvPfa4* mutants. The data analysis revealed a high correlation between different biological replicates, indicating that the data are highly reliable (Fig. S9). In addition, the heatmap also showed the data consistency between different sample replicates (Fig. 3E). Comparing with the HWD-2, it was found that the ∆*UvPfa3* treatment has 160 upregulated targets, while ∆*UvPfa4* treatment has 142 upregulated targets (Fig. 3A and B). KEGG enrichment analysis indicated that the downregulated targets of ∆*UvPfa3* were mainly enriched in pathways such as ABC transporter, fatty acid biosynthesis, TCA cycle and autophagy, and MAPK signaling pathway, and the downregulated targets of ∆*UvPfa4* were mainly enriched in pathways such as pentose phosphate, glycolysis, propionic acid metabolism, autophagy, and MAPK signaling pathways (Fig. 3C and D). Interestingly, we also found that the substrates of the two palmitoyltransferases were mainly located in the plasma membrane, cytoplasm, mitochondria, and nucleus (Fig. 3F).

### S-palmitoylation of MAP kinase UvSlt2 by palmitoyltransferase UvPfa4 modulates kinase phosphorylation

**Fig 4:**
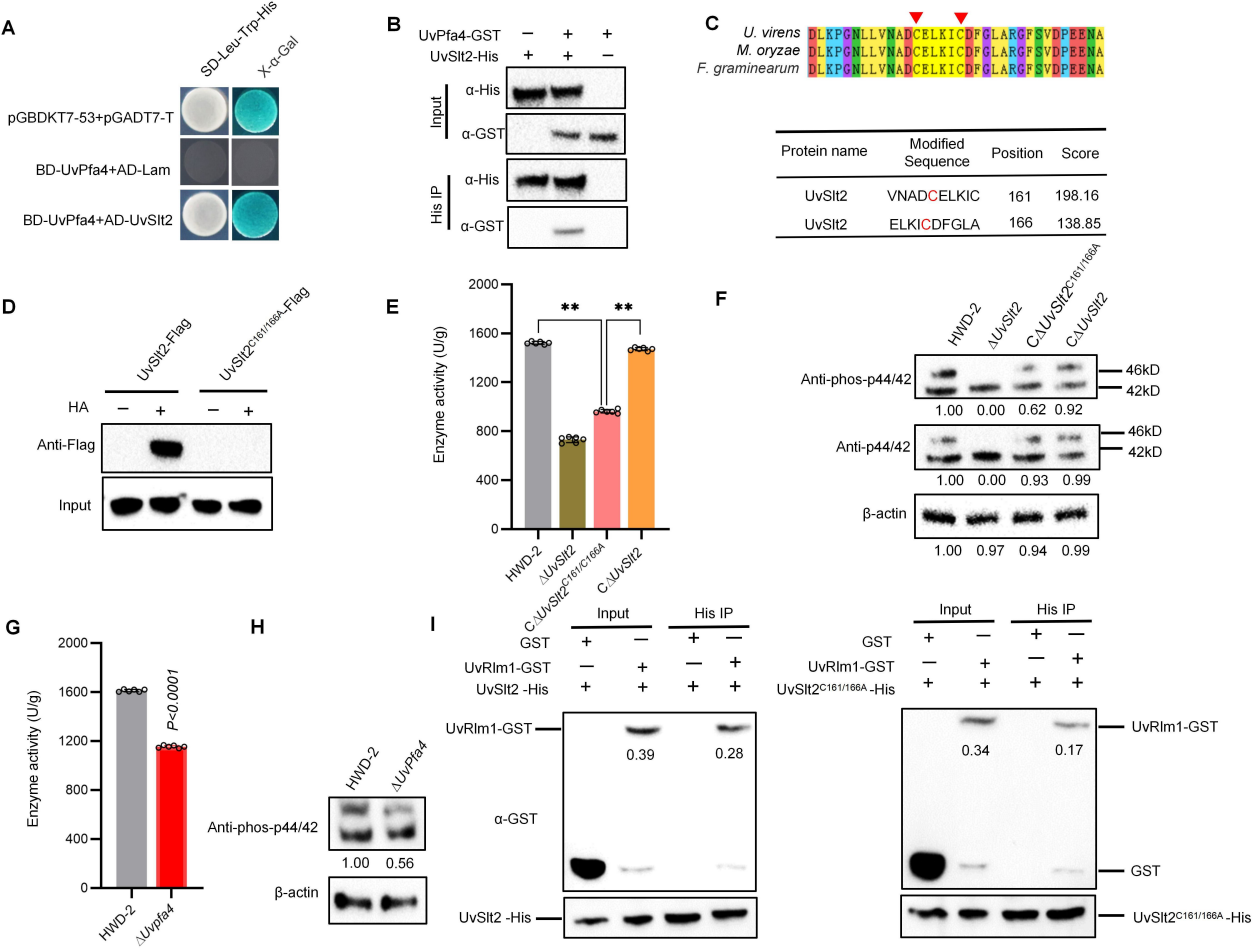
S-palmitoylation of MAP kinase UvSlt2 by palmitoyltransferase UvPfa4 modulates kinase phosphorylation. (**A**) Y2H analysis of the interaction between UvPfa4 and UvSlt2. pGBKT7-53 + pGAD7-T was the positive control; BD-UvPfa4 + AD was the negative control. BD, pGBKT7; AD, pGADT7. (**B**) GST pull-down assay showing the interaction between UvPfa4-GST and UvSlt2-His. Recombinant UvPfa4-GST or GST-bound resin was incubated with *E. coli* crude extracts containing UvSlt2-His and analyzed by immunoblotting. (**C**) Tandem mass spectrometry spectrum of *in vivo* S-palmitoylation sites of UvSlt2. (**D**) Western blot analysis of proteins extracted from the hyphae of UvSlt2-flag and UvSlt2^C161/166A^-flag cultured in PSB after HA treatment. (**E**) Palmitoylation of C161 and C166 affected the MAPK enzymatic activity of UvSlt2. The MAPK activity of the wild type, ∆UvSlt2, CUvSlt2^C161/166A^, and C∆UvSlt2 strains was detected. Data were collected from three independent experiments for each treatment and analyzed by Fisher’s test of least significant difference (LSD). Asterisks represent significant differences between the mutants and the wild-type HWD-2 by LSD at *P* < 0.05. (**F**) Western blot analysis of proteins extracted from PSB-cultured hyphae of the HWD-2, ∆UvSlt2, C∆UvSlt2^C161/166A^, and C∆UvSlt2 strains using anti-p42/44 antibody or anti-phos-p42/44 antibody. Total protein levels were visualized with anti-β-actin antibody, and the relative signal intensity of each band is indicated, with the HWD-2 sample set to 1.00. (**G**) The absence of UvPfa4 affected the MAPK enzymatic activity. The MAPK activity of the wild-type and ∆*UvPfa4* strains was detected. Data were collected from three independent experiments for each treatment and analyzed by Fisher’s test of least significant difference (LSD). Asterisks represent significant differences between the mutants and the wild-type HWD-2 by LSD at *P* < 0.05. (**H**) Western blot analysis of proteins extracted from PSB-cultured hyphae of the HWD-2 and ∆*UvSlt2* strains using anti-phos-p42/44 antibody. Total protein levels were visualized with anti-β-actin antibody, and the relative signal intensity of each band is indicated, with the HWD-2 sample set to 1.00. (**I**) *In vitro* pull-down quantification experiments by incubating equal amounts of proteins UvSlt2-His and UvSlt2^C161A/C166A^-His with UvRlm1-GST protein, separately. Recombinant UvRlm1-GST or GST-binding resin was incubated with crude extracts of *Escherichia coli* containing UvSlt2^C161A/C166A^-His and UvSlt2-His, separately, and the results were analyzed by immunoblotting.

To investigate how S-palmitoylation modification regulates the pathogenicity of *U. virens*, we selected six virulence-related proteins (Uv8b_02394, Uv8b_06341, Uv8b_07294, Uv8b_02457, Uv8b_02989, and Uv8b_01077) in MAPK and autophagy pathways from downregulated proteins of S-palmitoylation-modified proteomics of ∆*UvPfa3* and verified their interaction with palmitoyltransferase UvPfa3. We also selected five virulence-related proteins (Uv8b_01827, Uv8b_02989, Uv8b_06169, Uv8b_00381, and Uv8b_06314) in MAPK and autophagy pathways from downregulated proteins of S-palmitoylation-modified proteomics of ∆*UvPfa4* and verified their interaction with palmitoyltransferase UvPfa4 (Fig. S10A and B). We confirmed the interaction between UvPfa4 and UvSlt2 by yeast two-hybrid assay (Fig. 4A). We then performed *in vitro* pull-down assays with recombinant UvPfa4-GST (glutathione S-transferase) and UvSlt2-His (His tag) proteins purified from *E. coli*. We detected UvPfa4-GST from protein samples pulled down with UvSlt2-His loaded onto NTA-Ni beads (Fig. 4B), suggesting that UvSlt2-His and UvPfa4-GST interacted *in vitro*.

To further explore the roles of S-palmitoylation on UvSlt2 in plant pathogenic fungi, we performed site-directed mutagenesis of the S-palmitoylation sites in UvSlt2, which were located at C161 and C166 (Fig. 4C). To confirm that C161 and C166 are indeed the S-palmitoylation sites, we generated an UvSlt2C161A/C166A point mutant strain, in which the cysteine in the S-palmitoylation site was mutated to alanine (C-A). Sequences encoding normal and C-A UvSlt2 were fused with Flag, and the resulting constructs were expressed in the ΔUvSlt2 mutant. Immunoblotting of the Flag-enriched fraction indicated that S-palmitoylation of UvSlt2 was completely abolished by this mutation (Fig. 4D), confirming that UvSlt2 was S-palmitoylated at the two sites. To test whether palmitoylation could regulate the enzymatic activity of UvSlt2, we measured the enzyme activity of the double point mutant strain UvSlt2^C161A/C166A^ and found that the enzymatic activity of the double point mutant strain UvSlt2^C161A/C166A^ was significantly reduced (Fig. 4E). We performed western blot assays with an anti-p42/44 MAPK-specific antibody pairs and the results showed that the protein expression of UvSlt2 was not detected for the UvSlt2 mutant and that mutations of C161 and C166 in UvSlt2 did not cause decreased protein expression of UvSlt2. Since the enzymatic activity of the phosphorylated kinase affects the degree of phosphorylation, we then performed western blot assays with anti-p42/44 MAPK-specific phosphorylation antibody pairs, and the results showed that mutations of C161 and C166 in UvSlt2 decreased the UvSlt2 phosphorylation (Fig. 4F).

MAPK enzyme activity and UvSlt2 phosphorylation in the ∆*UvPfa4* mutant were also measured. Compared with the wild-type HWD-2, the MAPK enzyme activity in the ∆*UvPfa4* mutant was significantly reduced (Fig. 4G). Western blot analysis using anti-p42/44 MAPK-specific phosphorylation antibody showed that UvSlt2 phosphorylation was reduced in the ∆*UvPfa4* mutant (Fig. 4H).

Palmitoylation of proteins is a post-translational modification that plays an important role not only in functional regulation but also in regulating protein transport and membrane localization. We conducted protein analysis on the localization of UvSlt2. Subsequently, cytoplasmic and nuclear protein extraction was performed on the UvSlt2^C161A/C166A^-flag overexpression strains and the UvSlt2-flag overexpression strains, separately. The western blot results showed that equal amounts of UvSlt2 protein were detected in the cytoplasm and nucleus of UvSlt2^C161A/C166A^-flag overexpression and UvSlt2-flag overexpression strains, indicating that S-palmitoylation modification did not alter the localization of UvSlt2 (Fig. S12).

### S-palmitoylation on UvSlt2 is essential for fungal virulence

**Fig 5:**
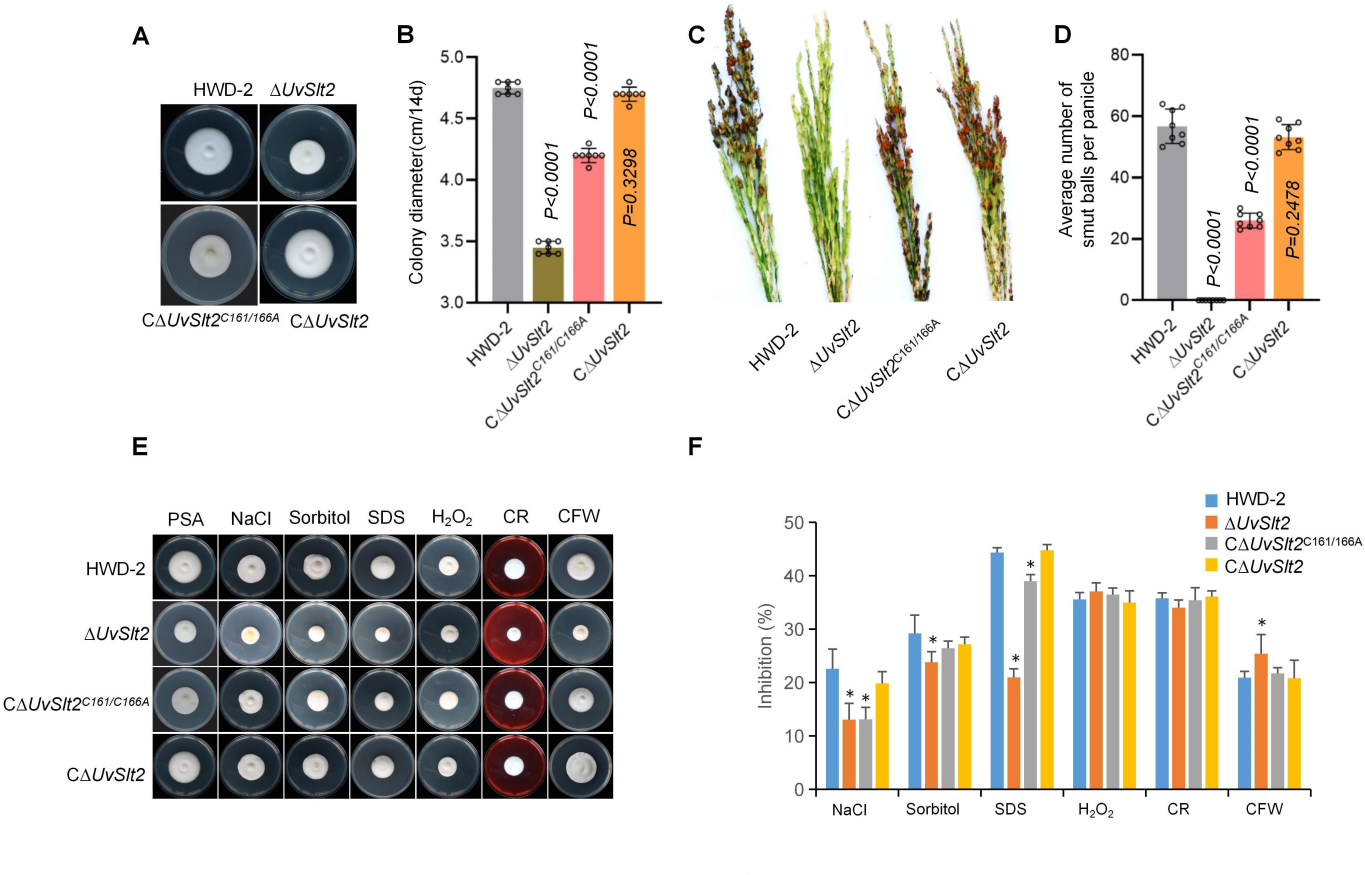
S-palmitoylation on UvSlt2 is essential for fungal development and virulence. (**A**) Colony morphology of *U. virens* wild-type HWD-2, ∆*UvSlt2*, C∆*UvSlt2^C161/166A^*, and C∆*UvSlt2* strains on PSA after 14 days of darkness at 28°C. (**B**) Colony diameters of the mutant strains on PSA medium after 14 days at 28°C. (**C**) Virulence assays of *U. virens* palmitoyltransferase family mutants on rice spikelets at 21 dpi. (**D**) The mean number of rice smut balls per panicle. Data were collected from three independent experiments for each treatment. Eight rice panicles were inoculated per replicate. (**E**) Colony morphology of the wild-type and mutant strains under different stresses. (**F**) Inhibition of colony growth of the wild-type and mutant strains under different stresses. Asterisks indicate statistically significant differences compared to the wild type (*P* < 0.05).

To investigate the effect of S-palmitoylation on the biological functions of UvSlt2, we compared growth rates in PSA medium among the wild-type, knockout strain ΔUvSlt2, complementation strain UvSlt2 and double-point mutant (UvSlt2^C161A/C166A^). The UvSlt2^C161A/C166A^ strain showed reduced growth rate and sporulation compared to the complementation strain UvSlt2 or the wild type (Fig. 5A and B). The UvSlt2^C161A/C166A^ strain showed significantly reduced sporulation and virulence (Fig. 5C and D; Fig. S1C). Subsequently, we observed the infection process of HWD-2 strain, ∆*UvSlt2* mutant, ∆*UvSlt2^C161A/C166A^* mutant, and C∆*UvSlt2* complementary strain in rice filaments and found that the hyphae of ∆*UvSlt2* mutant did not colonize the surface of rice filaments at 6 dpi, whereas the hyphae of HWD-2 strain, ∆*UvSlt2^C161A/C166A^* mutant strain, and C∆*UvSlt2* complementary strain colonized the surface of rice filaments at 6 dpi (Fig. S7B). The UvSlt2^C161A/C166A^ strain exhibited less sensitivity to the addition of NaCl or SDS in PSA growth medium (Fig. 5E and F). These results indicated that S-palmitoylation played a key role in UvSlt2 infection of *U. virens*.

### Mutations of S-palmitoylation sites impair binding between the UvSlt2 kinase and substrates

**Fig 6:**
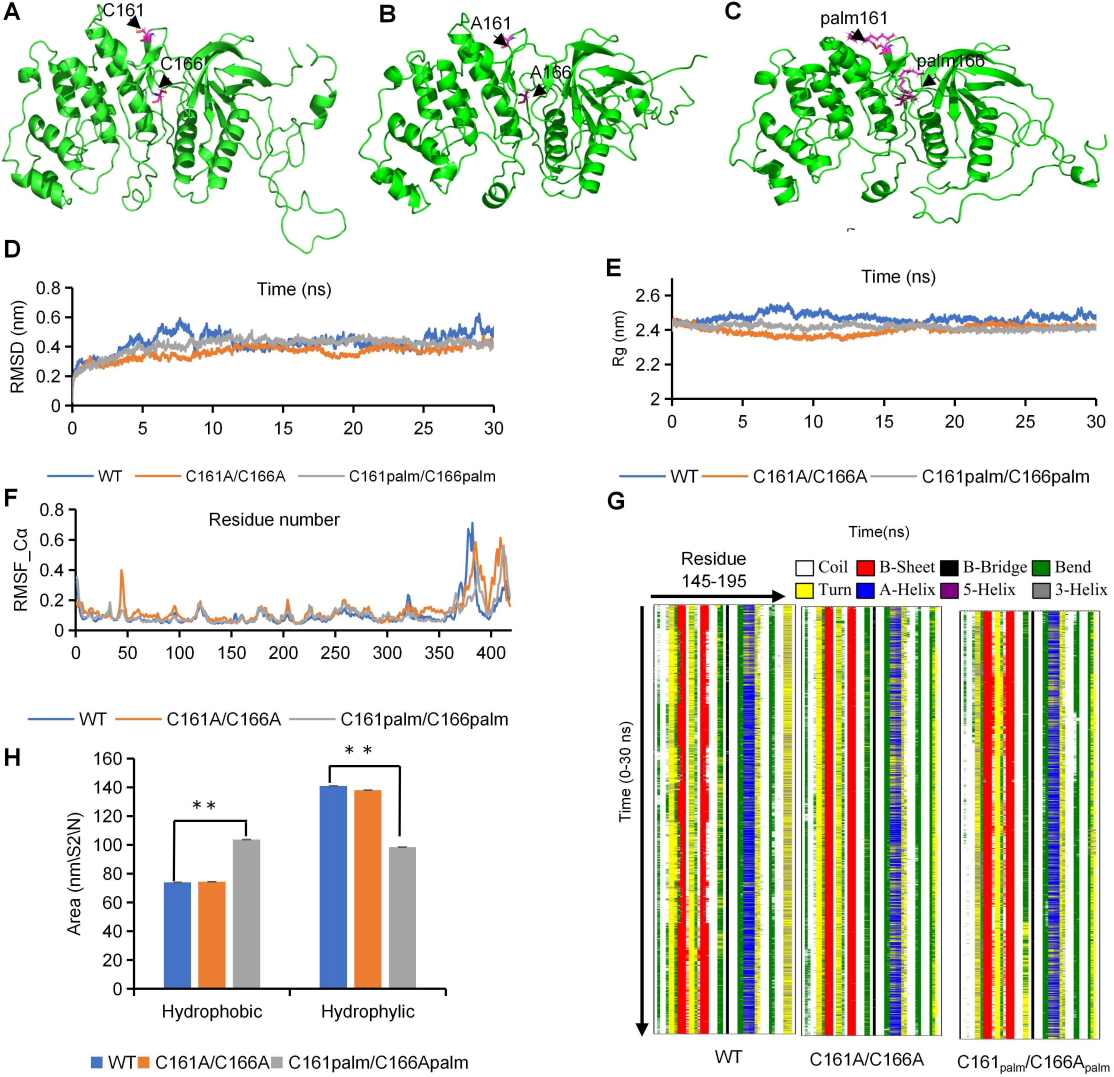
Mutations of S-palmitoylation sites impair binding between the UvSlt2 kinase and substrates. (**A**) Final structure of the simulations of non-S-palmitoylated UvSlt2. (**B**) Final structure of the simulations of UvSlt2^C161A/C166A^. (**C**) Final structure of the simulations of UvSlt2 with S-palmitoylation at C161 and C166. (**D-F**) Analysis of the root mean square deviations (RMSDs), root mean square fluctuations (RMSFs), and radius of gyrations (Rgs) for the different UvSlt2 systems. Trajectories of the overall RMSDs, radius of gyration profiles, and residue-specific RMSF profiles of the different UvSlt2 systems. (**G**) Secondary structure evolution of UvSlt2 and its mutants. The evolution in secondary structure at each frame was monitored using the dictionary of protein secondary structure (DSSP) algorithm. In the stripes, each pixel represents the secondary structure (color-coded) of a residue (145–195, x-dimension) at a given time in simulation (y-dimension). (**H**) The time evolution of the solvent-accessible surface (SAS) area was calculated for the different systems that were simulated. Asterisks indicate statistically significant differences compared to the wild type (*P* < 0.05).

To gain insights into potential mechanisms by which S-palmitoylation could affect UvSlt2 kinase phosphorylation, we first used simulations of molecular dynamics (MD) to determine how S-palmitoylation of C161 and C166 influences the structure of UvSlt2. When the cysteine residues at positions 161 and 166 were mutated to alanine, we found that the protein structures were almost the same as the unmodified UvSlt2 structure (Fig. 6A and B). The MD simulations of the S-palmitoylated UvSlt2 (C161_palm_/C166_palm_) indicated that S-palmitoylation of C161 and C166 resulted in structures that were different from the unmodified UvSlt2 (Fig. 6C). The structures of the S-palmitoylated (C161_palm_/C166_palm_) vs the unmodified UvSlt2 were significantly different, whereas the A mutants (C161A/ C166A) were nearly identical to the unmodified UvSlt2 during the entire course of simulations (Fig. 6D through F; Table S5). The simulations revealed that the mutants and unmodified UvSlt2 shared similar secondary structures and underwent minor changes from start to final states (Fig. 6G). Because of the perturbation of the secondary structures and the conformational fluctuations, the structures of S-palmitoylation UvSlt2 had a strong tendency to form a hydrophobic solvent-accessible surface area (Fig. 6H; Table S5). When cysteine was mutated to alanine, the hydrophobic solvent-accessible surface area did not change. After UvSlt2 S-palmitoylation, the hydrophobic solvent-accessible surface area increased and thereby affected binding between the UvSlt2 enzyme and substrates. To verify the binding affinity of UvSlt2 to its substrates, we selected a candidate protein UvRlm1, which has been reported as a substrate for UvSlt2 homologous protein in *Saccharomyces cerevisiae* (27), and conducted *in vitro* pull-down experiments using purified recombinant UvRlm1-GST and UvSlt2-His proteins from *Escherichia coli*. We detected UvRlm1-GST in protein samples loaded with UvSlt2-His onto NTA Ni magnetic beads, indicating an interaction between UvSlt2-His and UvRlm1-GST *in vitro*. Then, we conducted *in vitro* pull-down quantification experiments by incubating equal amounts of proteins UvSlt2-His and UvSlt2^C161A/C166A^-His with UvRlm1-GST protein, separately. The results demonstrated that the amount of UvRlm1-GST protein detected in the protein samples loaded with UvSlt2^C161A/C166A^-His onto NTA Ni magnetic beads was weaker than that detected in the protein samples loaded with UvSlt2-His onto NTA Ni magnetic beads (Fig. 4I). Compared to the HWD-2 strain, the ∆*UvRlm1* mutant had a lower growth rate and complete loss of pathogenicity (Fig. S11A through D). In rice filaments, hyphae of the ∆*UvRlm1* mutant strain did not colonize the surfaces of rice filaments at 6 dpi (Fig. S11E). These results indicated that the UvPfa4-UvSlt2-UvRlm1 module has a positive regulatory effect on the virulence of *U. virens*.

## DISCUSSION

Precursor proteins undergo protein post-translational modification by binding different small molecule biochemical groups (such as phosphates, acetates, lipids, and carbohydrates). This effectively changes the structural characteristics and physical and chemical properties of protein macromolecules, endowing proteins with strong functional activity, and resulting in the transition from precursor protein to functional protein (28). With the discovery of novel post-translational modifications, new data suggest that various post-translational modifications in eukaryotes are related to cellular physiology and pathogenesis. Palmitoylation covalently attaches palmitate to cysteine residues of proteins through thioester bonds, regulates protein trafficking, promotes protein complex formation, and alters cellular localization under normal physiological and pathological conditions (29–32). The functions and regulatory mechanisms of palmitoylation have been extensively investigated in humans, animals, and plants. In the process of pathogenic infection of plants, the role of palmitoylation has been reported in plant disease resistance. In *Arabidopsis thaliana*, S-palmitoylation affected eATP-induced phosphorylation protein kinase activity and negative regulation of plant natural immune signal transduction and triggered immune signal transduction induced by the ligand-receptor kinase complexes formed (33). NB-LRR protein is modified by S-palmitoylation to change its cytoplasmic membrane localization and improve the speed of response to pathogenic infection, thereby mediating the resistance of *Arabidopsi*s (14). De-S-palmitoylation of receptor-like kinase PBL19 promotes massive nuclear translocation and triggers plant immune responses (15). However, little is known about palmitoylation in pathogenic fungi. This study is the first to report the pathogenic role and regulation mechanisms of S-palmitoylation in a pathogen.

In this study, we used the acyl-biotin exchange method to detect S-palmitylation modification because commercial antibodies for S-palmitylation are not available. The acyl-biotin exchange method has become a common method to detect protein post-translational modifications. The essence of the acyl-biotin exchange method is to convert the post-translational modification to the acylated biotin group. In humans, the acyl-biotin exchange (ABE) assay combined with a bio-orthogonal palmitic acid probe is used to detect palmitoylation of renal tissue proteins by click chemistry and the presence of palmitoylation modification at the C-terminus of the MyoD family inhibitory protein MDFIC was confirmed (34, 35). In animals, it was found that the NLRP3 ectopic expression was acetoxylation as revealed by acyl biotin exchange (36). In plants, OsCBL2 and OsCBL3 S-palmitylation by OsDHHC30 were demonstrated by acyl-biotin exchange and acyl-PEG exchange assays, respectively, in the cell endomembrane system (37). This leads us to use the acyl-biotin exchange assay to detect palmitoylation modifications in fungi. In this study, we investigated the Gene Ontology (GO) functional classification of all S-palmitoylation-modified proteins. Enrichment analysis for biological processes indicated that the set of S-palmitoylated proteins was significantly enriched in metabolic and cellular processes, suggesting a significant role for S-palmitoylation in metabolism. For molecular functions, S-palmitoylated proteins were the most enriched in binding, in agreement with the highly enriched metabolic processes in biological processes mentioned above (Fig. S8C; Table S3). These findings suggested that S-palmitoylation may be involved in the basic life activities of *U. virens*. Notably, palmitoylation plays an important role in many biological processes, such as protein signaling, establishment of cell polarity, cell proliferation, and differentiation. Therefore, this study provides a basis for further understanding the regulatory mechanisms of palmitoylation in plant pathogenic fungi.

The irreversible inhibitor 2 BP has been used as a pharmacological tool to study protein palmitoylation. 2 BP can effectively inhibit the S-palmitoylation of PD-L1 in tumor cells, thereby inhibiting tumor growth. 2 BP inhibits S-palmitoylation of the NSP1 protein, thereby weakening chikungunya viral infection. 2 BP inhibited S-palmitoylation in HCT116 cells, promoted the autophagic degradation of NOD2 mediated by SQSTM1/p62, and inhibited NOD2-mediated inflammation (12, 38, 39). In this study, we found that treatment of *U. virens* with S-palmitoylation inhibitor 2 BP is essential for the growth, development, and infection processes of *U. virens,* which demonstrated that 2 BP can also act as an inhibitor of protein palmitoylation modification of *U. virens*. Due to the effective prevention of *U. virens* by 2 BP inhibitor, 2 BP inhibitor could be chemically modified to promote inhibition activity. Therefore, palmitoacylation-related inhibitors have the potential to be developed as fungicides for the prevention and control of rice false-smut disease.

In biological processes among eukaryotes, PATs are important protein post-translational modification enzymes in plants and animals, which play key roles in protein palmitoylation. Comparison with yeast DHHC family members revealed that *U. virens* has six DHHC family members. This is consistent with the catalytic domains of palmitoyltransferases proteins reported in yeast and *Arabidopsis* (4, 40). In *U. virens*, there are 2–4 transmembrane domains, as opposed to 4–6 transmembrane domains in yeast (1), suggesting that the functions of palmitoyltransferases might not be the same in different species. In *U. virens*, deletion of five palmitoyltransferases led to various phenotypic changes. We tried to knock out the UvErt2 gene many times. Unfortunately, we could not obtain the UvErt2 knockout mutant successfully. Thus, UvErt2 might be a lethal gene in *U. virens*. It is speculated that different palmitoyltransferases affect diverse pathways by modifying different substrates, and thus regulate the biological phenotypes of *U. virens*.

**Fig 7:**
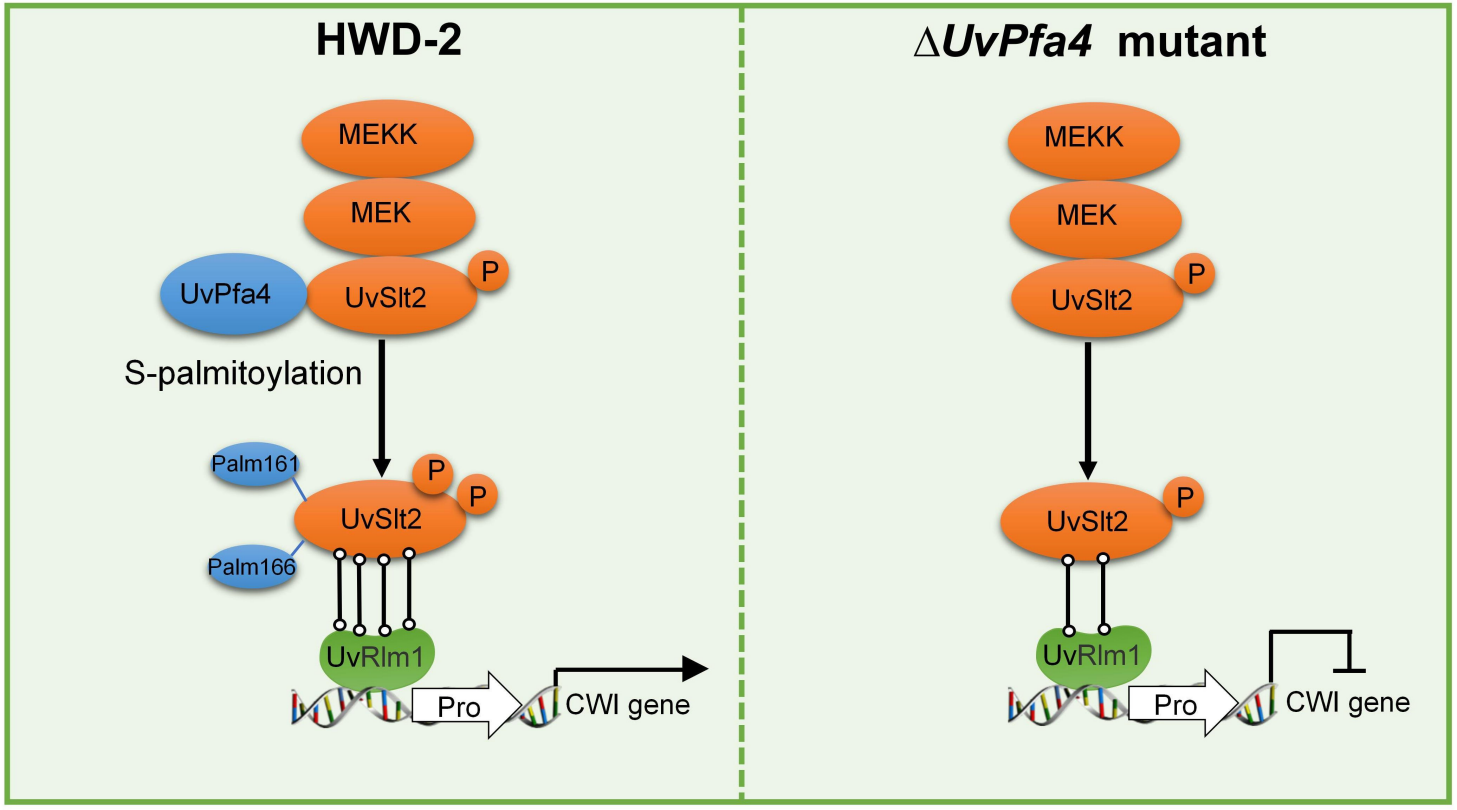
A working model illustrating how S-palmitoylation promotes *U. virens* virulence during rice infection. (Left) S-palmitoylation promotes *U. virens* virulence through palmitoylating MAP kinase UvSlt2 by palmitoyltransferase UvPfa4 and enhancing phosphorylation levels of the kinase, thereby increasing hydrophobic solvent-accessible surface area and binding activity between the UvSlt2 enzyme and its substrate UvRlm1. (Right) After infection by the ∆*UvSlt2^C161/166A^* mutant, phosphorylation levels of the kinase were significantly reduced, resulting in interference with the substrate UvRlm1 binding activity.

Protein phosphorylation and de-phosphorylation play important roles in biological signal recognition and transduction, which are universal mechanisms in living organisms. Mitogen-activated protein kinase (MAPK) is a common type of protein kinase in eukaryotes and is most closely related to signal transduction (41). Previous studies showed that Slt2 could phosphorylate and activate the expression of Rlm1 in *Pneumocystis carinii* (42). Slt2 phosphorylates Rlm1 and is required for virulence of *Aspergillus flavus* (43). In *Saccharomyces cerevisiae,* Slt2 regulates the CWI pathway by phosphorylating and activating the transcriptional activity of Rlm1 (44, 45). In this study, we identified the S-palmitoylation modified protein sites of *U. viren*s, as well as quantitative proteomics analysis of S-palmitoyltransferases ∆*UvPfa3* and ∆*UvPfa4* mutants, and confirmed that UvSlt2 was S-palmitoylated by the palmitoyltransferase UvPfa4. Point mutation of the S-palmitoylation site of UvSlt2 significantly decreased its enzymatic activity and altered phosphorylation by the Slt2-MAPK pathway to regulate the virulence of *U. virens*. Indeed, the Slt2-MAPK pathway plays a conserved role in the pathogenesis of filamentous fungal pathogens (46). We hypothesized that S-palmitoylation promotes *U. virens* virulence through palmitoylating MAP kinase UvSlt2 by palmitoyltransferase UvPfa4 and enhancing phosphorylation levels of the kinase, thereby increasing hydrophobic solvent-accessible surface areas and binding activity between the UvSlt2 enzyme and its substrate UvRlm1 (Fig. 7). This study provides a new theoretical basis for post-translational modification in the pathogenic mechanisms of and disease strategies for plant pathogenic fungi.

## MATERIALS AND METHODS

### Fungal strains and culture

The *U. virens* strain HWD-2 was used as the reference wild-type strain. The HWD-2 and transformed strains derived from HWD-2 were maintained and cultured on PSA at 28°C under dark conditions. After shaking at 180 rpm for 7 days, the PSB cultures were homogenized in a blender to prepare mycelial and spore suspensions.

### Vegetative growth, conidiation and virulence assays

For vegetative growth, mycelial plugs were transferred from 14-day-old PSA plates and grown on fresh PSA medium at 28°C. After 14 days of incubation, the radial growth of vegetative mycelia was measured. For conidial production, strains were grown in PSB medium at 28°C. After shaking at 180 rpm for 7 days, the culture was ﬁltered through four layers of gauze to collect spores, and conidial concentrations were adjusted to 10^6^/mL. Conidial length and width were measured under a microscope with at least 100 conidia per replicate. Each test treatment was repeated three times. Rice plants (cv. Wanxian-98) were inoculated with 2 mL suspension by syringe in the middle section of distal internodes at the eighth stage of panicle development. The treated rice plants were placed in a greenhouse with a relative humidity of 95% ± 5% and a temperature of 26% ± 2°C. Under these conditions, the rice plants were cultivated for 21 days, and the numbers of diseased spikelets and smut balls per panicle were counted. The experiment was repeated three times. After inoculation of rice panicles with *U. virens*, samples were collected after 1 and 6 days and stored in 2.5% glutaraldehyde ﬁxative and then used to observe the infection process by scanning electron microscopy.

### Protein extraction and digestion

Mycelial samples were ground in liquid nitrogen and 600 µL SDT lysis buffer was added to each sample. This mixture was sonicated in a boiling water bath for 3 min. After this mixture was centrifuged at 16,000 × *g* at 15°C, samples were quantified for protein using the BCA method. Twenty micrograms of protein from each sample was subjected to SDS-PAGE gel electrophoresis. Each sample was added with N-ethylmaleimide (NEM) (total concentration 0.1 M) at 4°C overnight (free sulfhydryl group on labeled Cys). The excess NEM was removed by TCA-acetone precipitation, and the protein precipitate was re-dissolved in 1 mL phosphate-buffered saline (PBS; pH 7.4) containing 6 M urea and 2% SDS. Samples were incubated for 1.5 hours at 25°C with 3 mL of 1 M hydroxylamine (HA; pH 7.4), 1 mL PBS, 5 mM EDTA, and 500 µL of 4 mM biotin-HPDP dissolved in DMSO. The protein of each sample was concentrated to 150 µL with a 5K ultrafiltration tube, precipitated overnight with 6× cold acetone, washed, and dried. A total of 150 µL of 50 mM ammonium bicarbonate (ABC) was added, an appropriate amount of trypsin was added, and enzymolysis was conducted at 37°C overnight.

### Fraction and enrichment of cysteine S-palmitoylation peptides

Peptides were dissolved in 200 µL loading/washing buffer (0.2% SDS, 0.2% Triton X-100, and 500 mM NaCL), and 100 µL of high-capacity streptavidin beads was added into the sample tube. Then the tube was vortexed and reacted in the dark for 2 h. Non-specific adsorbed proteins were washed away. Palmitoylation peptides were eluted with 0.5 mM Tris(2-carboxyethyl)phosphine (TCEP), and after desalination, samples were dissolved in 0.1% formic acid.

### LC-MS/MS analysis

The samples were separated by chromatography using a nanoliter Easy nLC 1200 chromatographic system (Thermo Scientific). The column was equilibrated with 95% solution A (0.1% formic acid aqueous solution). After injection into the Trap Column (100 µm × 20 mm, 5 µm, C18, Dr. Maisch GmbH), the samples were separated by gradient through the chromatographic column (75 µm × 150 mm, 3 µm, C18, Dr. Maisch GmbH). Peptides were separated and analyzed by data-dependent acquisition (DDA) mass spectrometry on a Q-Exactive HF-X mass spectrometer (Thermo Scientific).

### Bioinformatic analyses

The acquired MS/MS data were tested with MaxQuant software (version 1.5.2.8) against the UniProt_*U.virens* protein database (https://www.ncbi.nlm.nih.gov/nuccore/JHTR00000000). Trypsin/P was specified as a cleavage enzyme allowing a maximum of two missing cleavage sites. The mass tolerance for precursor ions was set to 20 ppm in the first search and 5 ppm in the main search, and the mass tolerance for fragment ions was set to 0.02 Da. FDR thresholds for peptides, proteins, and modification sites were less than 1%. Cysteine peptides were considered as false positives and removed from the list with any of the following conditions: when peptides were identified from reverse or contaminant protein sequences; when peptides had a score below 40; when site localization probability was below 0.75; or when sites were mapped to the C terminus of the peptide unless the peptide C terminus was the end of the corresponding protein. GO annotation and protein–protein interaction analyses were performed using DAVID software (the Database for Annotation, Visualization and Integrated Discovery) and STRING (version 10.5). The sequence logo representations of significant motifs were identified and generated by Motif-X software (version 5.0.2). Pathways were classified into hierarchical categories according to the KEGG website. WoLF PSORT (version 0.2, https://wolfpsort.hgc.jp) was used to predict protein subcellular localization.

### RNA extraction and RT-qPCR

Total RNA was extracted using the TRIzol reagent (Vazyme Biotech, Nanjing, China). First-strand cDNA synthesis was carried out with a cDNA Synthesis SuperMix (ABconal, China). RT-qPCR was performed with TransStart Tip Green qPCR SuperMix (ABconal, China). Transcript levels were normalized using the *U. virens* reference gene β-tubulin gene (Uv8b_900). The experiment was repeated three times.

### Gene deletion and complementation

To generate gene deletion mutants, 1 kb of upstream and downstream flanking sequences of the gene were ligated with pGKO to generate the final deletion vector. The construct was inserted into *A. tumefaciens* strain EHA105, and then transformed into conidia of wild-type HWD-2. Hygromycin-resistant transformants were isolated and subsequently screened by PCR with primers listed in Table S6. For complementation assays, a native promoter region and a full-length *UvPfa3*, *UvPfa4*, *UvPfa5*, *UvAkr1,* or *UvSwf1* were ligated to construct complementary vectors. The EHA105 strain with the complementation vectors was transformed through *A. tumefaciens*-mediated transformation by co-culture with conidia of the gene deletion mutant. Transformants were selected on PSA supplemented with 1 mg mL^–1^ antibiotic G418, and screened by PCR with the primers listed in Table S7.

### Yeast two-hybrid assay

For interaction analysis of *UvSlt2* and *UvPfa4* in yeast, the coding sequences of *UvPFA4* and *UvSLT2* were cloned into pGBKT7 and pGADT7, respectively. The bait vector pGBKT7-*UvPfa4* and the prey vector pGADT7-*UvSlt2* were co-transformed into yeast strain Y2HGold (Coolaber, China). Positive clones were selected on SD–Trp–Leu–His (SD-3) agar (Coolaber, China), and further confirmed on SD–Trp–Leu–His (SD-3) agar (Coolaber, China) containing X-α-Gal (Coolaber, China). The interaction between pGBKT7-53 and pGADT7-T was used as the positive control, and that of pGBKT7-*UvPfa4* and pGADT7 was used as the negative control.

### GST pull-down assays

The coding sequences of *UvSlt2* and *UvPfa4* were cloned into vectors Pet32a and pGEX-4T-2, respectively. The constructs *UvSlt2*-Pet32a and *UvPfa4*-pGEX4T-2 were introduced individually into *E. coli* BL21 (DE3) cells for production and purification. The *UvPfa4*-GST fusion protein was extracted from *E. coli* cells and incubated with 100 µL of glutathione–agarose beads (MedChemExpress, China) at 4°C for 4 hours with shaking. After centrifugation at 4°C, the beads were collected and washed with PBS three times. Beads were then incubated with recombinant *UvSlt2*-His protein at 4°C for 2 h with shaking and then washed with PBS three times. Beads were boiled for 5 min at 100°C in 40 µL SDS sample loading buffer, and the proteins were analyzed by immunoblotting with anti-His (1:5,000, Biodragon, China) and anti-GST antibody (1:5,000, Biodragon, China).

### Site-directed mutagenesis and purification of UvSlt2

For site-directed mutagenesis assays, a native promoter region and a full-length *UvSlt2* were cloned into vector pCETNS-4-Flag. C161 and C166 were identified as potential S-palmitoylation sites of UvSlt2 protein. We mutated cysteine to glycine (TGC-GCC) and obtained a double-point mutation sequence by PCR reaction, and then this sequence was cloned into vector pCETNS-4-Flag. The EHA105 strain with the complementation vectors was transformed through ATMT by co-culture with conidia of the Δ*UvSlt2-1* mutant. Transformants were selected on PSA supplemented with 1 mg/mL antibiotic G418. Transformants (Δ*UvSlt2-1* complementation strain, C161A and C166A double-point mutation strain) were confirmed by western blotting using anti-Flag antibody. For immunoprecipitation (IP), total proteins were extracted from these strains after infiltration and incubated with Anti-Flag M2 affinity gel (Yeasen Biotech, China). The conjugated beads were washed three times with PBS and incubated with whole cell lysates overnight at 4°C. The beads were then washed three times with PBS to remove unbound proteins. Bound proteins were boiled in a SDS loading buffer for 5 min and used in western blotting.

### UvSlt2 activity assay

*UvSlt2* activity assay was measured using a MAPK ELISA Kit (Abbexa, UK). Double-antibody one-step sandwich enzyme-linked immunosorbent assay (ELISA) was used in this kit. Samples, standards, and HRP-labeled detection antibodies were added into the microwells which were pre-coated with MAPK antibody. The microwells were incubated and washed thoroughly with PBS buffer. Tetramethylbenzidine (TMB) was used as a chromogenic substrate where the intensity of the color is positively correlated with the mitogen-activated protein kinase in the sample. The absorbance (OD value) was measured with a microplate reader at 450 nm wavelength to calculate the sample activity. All determinations were repeated three times.

### Molecular dynamics simulations

The initial model of the UvPR1H module was prepared from the available crystal structure via structural modeling using MODELLER software (version 9.14). All simulations were based on the initial model and three systems were prepared for simulation using GROMACS (version 4.6) in conjunction with the OPLS-AA/L all-atom force field. The protein was then solvated in simple point charge (SPC) water molecules in a cubic box, with the box edges ~1.0 nm from any atom of the protein, and additional Na^+^ and Cl^−^ ions were added to neutralize the charge of each system. Each system was then energy minimized using the steepest descents integrator setting either until the maximum force was less than 1,000 kJ/mol/nm on any atom or until additional steps resulted in a potential energy change of less than 1 kJ/mol. Next, the simulations were performed under a constant temperature of 300 K, and the V-rescale algorithm was used with a temperature coupling time constant of 0.1 ps. All bond lengths were constrained using the linear constraint solver (LINCS) algorithm. Measurement of Van der Waals interactions used a simple cut-off at 1.4 nm, and long-range electrostatic interactions were handled using the particle mesh Ewald (PME) method with a fourth-order spline interpolation and a 0.1 nm Fourier grid spacing. Once each system was sufficiently equilibrated around the target temperature, isotropic pressure coupling was used and the constant pressure was set to 1.0 bar in all directions with a pressure coupling time constant of 1.0 ps. Finally, each system was subjected to 10 ns of MD simulation, and the time step used in the simulations was 2 fs. All analyses were performed using the GROMACS suite of tools and a secondary structure recognition algorithm (DSSP), which was implemented in GROMACS. The PyMOL Molecular Graphics System (version 1.7.2) was used to present the structural results of this study.

### Inhibition of protein S-palmitoylation with 2-BP

The S-palmitoylation inhibitor 2 BP was dissolved in DMSO and adjusted to concentrations of 75, 150, or 300 µM. The mycelia were separately treated with different concentrations of 2 BP and then incubated with shaking for 7 days. Total protein was extracted, and the S-palmitoylation of *U. virens* was measured by HRP Anti-Streptavidin (Beyotime Biotechnology, Shanghai China).

### Biotin-switch assay

The S-palmitoylated proteins were analyzed using the HRP Anti-Streptavidin kit (Aimsmass AM10315) based on the biotin-switch assay as described previously. Mycelia transiently transfected with flag-tagged proteins were homogenized in lysis buffer containing 50 mmol/L Tris (pH 7.5), 150 mmol/L NaCl, 0.5% (vol/vol) Triton-X 100, and 1 × protease inhibitor for 1 h on ice. After centrifugation at 20,000 × g for 15 min at 4°C, 25 mmol/L N-ethylmaleimide was added to the supernatant to block free sulfhydryl groups with gentle shaking overnight at 4°C. The proteins were precipitated with chloroform/methanol (1:3) and then divided into two parts. One part was treated with 800 µL of 1 mol/L HA in the presence of biotin-N-[6-(biotin amide) hexyl]-3′-(2′-pyridyl disulfide) propionamide (HPDP). The other fraction was treated with 800 µL of 1 mol/L Tris·HCl with the addition of biotin-HPDP, and incubated at room temperature overnight, followed by the addition of 1 mL methanol/chloroform (3:1, vol/vol) to precipitate the protein. All these steps were performed under indirect light. The protein pellet was dissolved in a wash buffer. The biotinylated proteins were then mixed with 30 µL of Streptavidin Magnetic Beads (Beyotime P2159) and incubated overnight at 4°C. The beads were washed three times with a washing buffer. In these steps, S-palmitoylated proteins could be purified with streptavidin beads, which bind biotin with very high affinity and specificity. The protein was then eluted and used for western blotting with flag-antibody (ABclonal AE092).

### Western blotting

Extracted proteins were re-dissolved in 8 M urea. Protein concentration was determined with a BCA kit (Solarbio, China) according to the manufacturer’s instructions. The proteins separated on SDS-PAGE gels were transferred onto a polyvinylidene fluoride membrane with a BioRad electroblotting apparatus. Subsequent transformants were subjected to western blotting analysis with p44/42 MAPK (Erk1/2) Antibody (Cell Signaling Technology, USA), and Phospho-p44/42 MAPK (Erk1/2) Antibody (Cell Signaling Technology, USA). The signals on the blots were detected using Pierce ECL Western blotting substrate (Thermo Fisher Scientific, USA) in ChemiDoc XRS+ system (Bio-Rad, USA).

## Data Availability

The mass spectrometry proteomics data have been deposited to the Proteome X change Data set (PRIDE) under accession numbers PXD048216 and PXD054433.

## SUPPLEMENTAL MATERIAL

Supplemental figures mbio.02704-24-s0001.pdf Fig. S1-S12.

Table S1 mbio.02704-24-s0002.xlsx Modification site information of S-palmitoylated proteins in *U. virens*.

Table S2 mbio.02704-24-s0003.xlsx Subcellular localization of S-palmitoylated proteins in *U. virens*.

Table S3 mbio.02704-24-s0004.xlsx Gene ontology enrichment of S-palmitoylated proteins in *U. virens*.

Table S4 mbio.02704-24-s0005.xlsx KEGG pathway enrichment of S-palmitoylated proteins in *U. virens*.

Table S5 mbio.02704-24-s0006.xlsx Data of molecular dynamics simulations of C161/166Palm affects the protein structure of UvSlt2.

Table S6 mbio.02704-24-s0007.xlsx PCR validation primers used for palmitoyltransferase gene knockout transformants of *U. virens*.

Table S7 mbio.02704-24-s0008.xlsx Primers used for construction of palmitoyltransferase gene complementation vectors of *U. virens*.

Table S8 mbio.02704-24-s0009.xlsx Differential geneprotein expression in quantitative proteomics of UvPfa3 and UvPfa4 S-palmitoylation modifications by UvPfa3 and UvPfa4.

Table S9 mbio.02704-24-s0010.xlsx S-palmitoylated proteins grouped as the major clusters in protein-protein interaction map involving in autophagy-related proteins, proteasome, endocytosis pathway proteins, autophagy-related proteins, ribosome structural constituents, and MAPK pathway proteins.

ASM does not own the copyrights to Supplemental Material that may be linked to, or accessed through, an article. The authors have granted ASM a non-exclusive, world-wide license to publish the Supplemental Material files. Please contact the corresponding author directly for reuse.
