# Supplementary material for: S-palmitoylation of MAP kinase is essential for fungal virulence
Source: mBio. 2024 Oct 29;15(12):e02704-24. doi: 10.1128/mbio.02704-24 (PMC11633104; doi:10.1128/mbio.02704-24)
Supplement: Supplemental figures — Fig. S1-S12. [file mbio.02704-24-s0001.pdf]

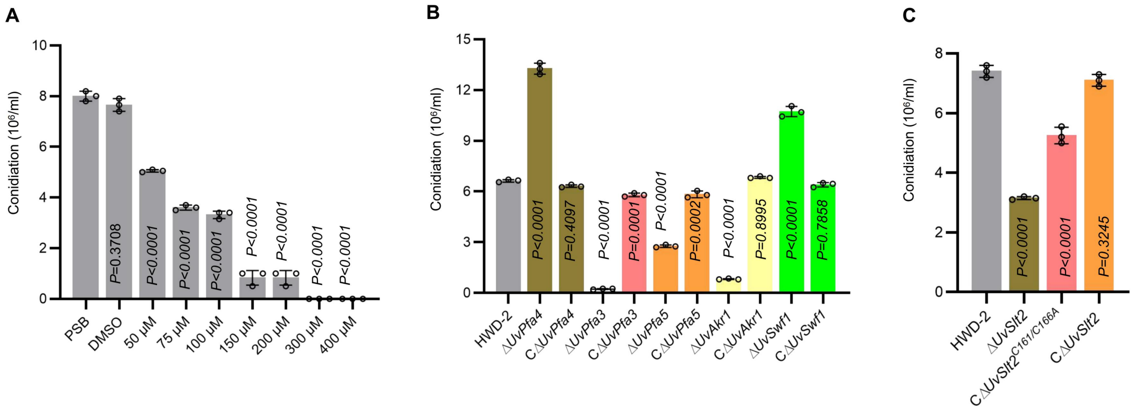

FIG S1 Conidiation of *U. virens* mutant strains. (A) Conidial production of the wild-type strain HWD-2 grown in PSB medium containing different concentrations of 2-BP at 180 rpm for 7 d. (B) Conidial production of the palmitoyltransferase family mutants grown in PSB medium for 7 d at 180 rpm. (C) Conidial production of the wild-type and point-mutated strains grown in PSB medium for 7 d at 180 rpm.

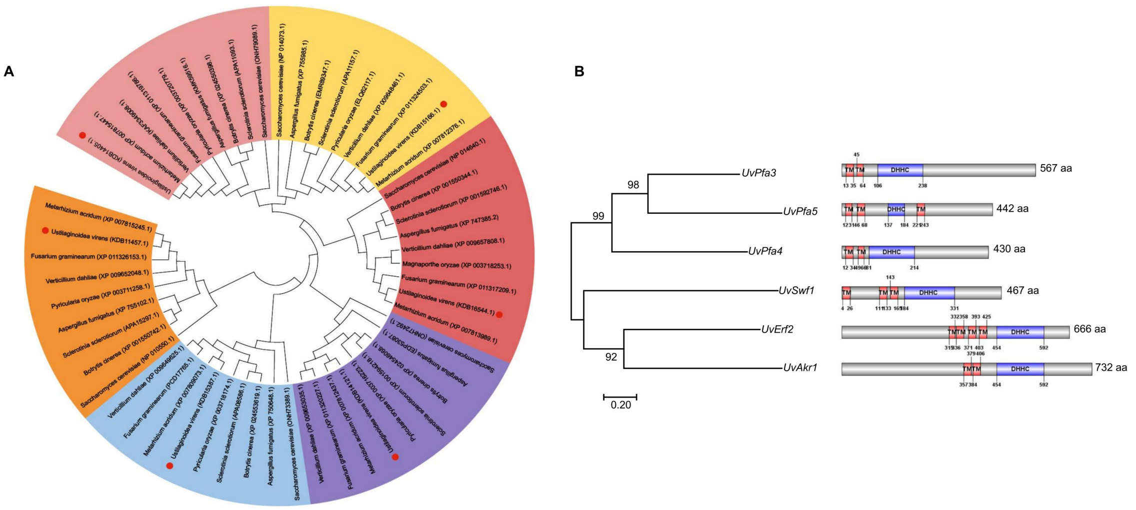

FIG S2 Identification of palmityltransferase family proteins in *U. virens*. (A) Neighbor-joining tree of palmityltransferase family protein homologs from different fungal genomes generated with MEGA7.0. The bootstrap percentage values from 1000 repeats are shown at the branch nodes. (B) Predicted Pfam domain of palmityltransferase family proteins.

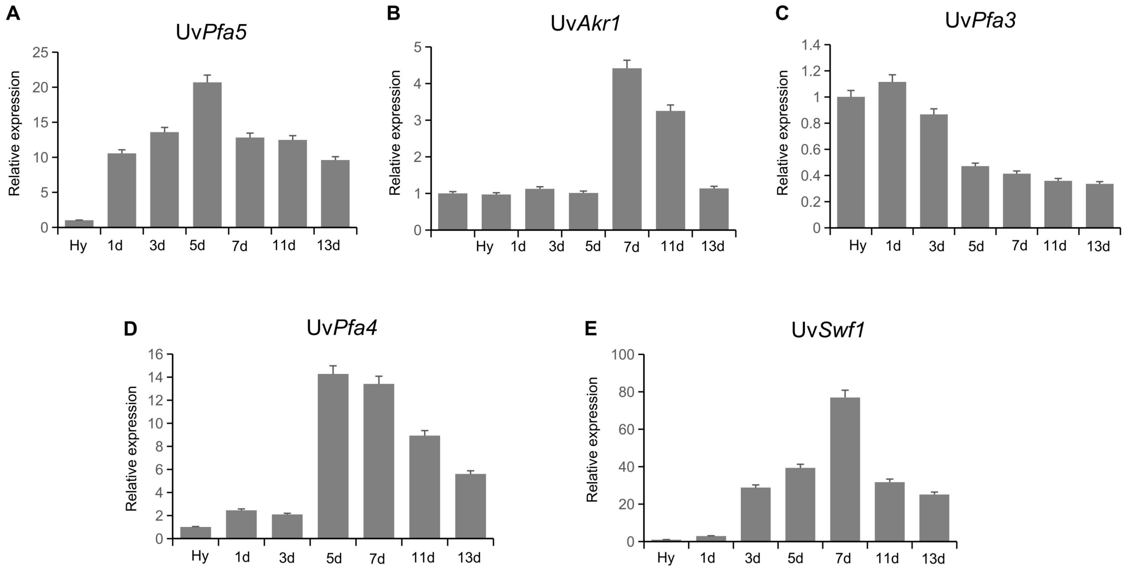

FIG S3 Expression of palmityltransferase family genes in *U. virens*. Expression profiles as determined by RT-qPCR of (A–E) UvPfa5, UvAkr1, UvPfa3, UvPfa4 or UvSwf1 genes relative to β-tubulin in *U. virens* hyphae grown in PSB or at different stages of infection on rice (1–13 d).

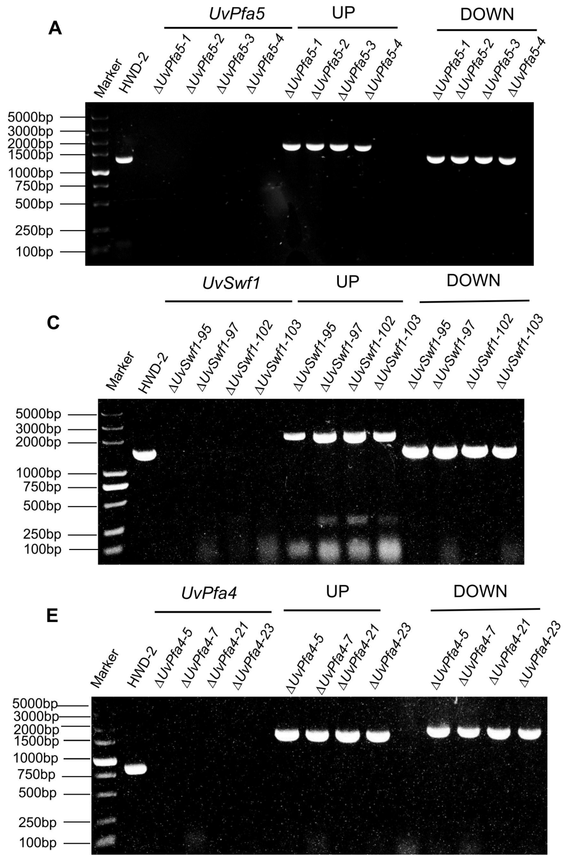

FIG S4 PCR identification of complementation mutants of (A–E) UvPfa5, UvAkr1, UvSwf1, UvPfa3 and UvPfa4 genes.

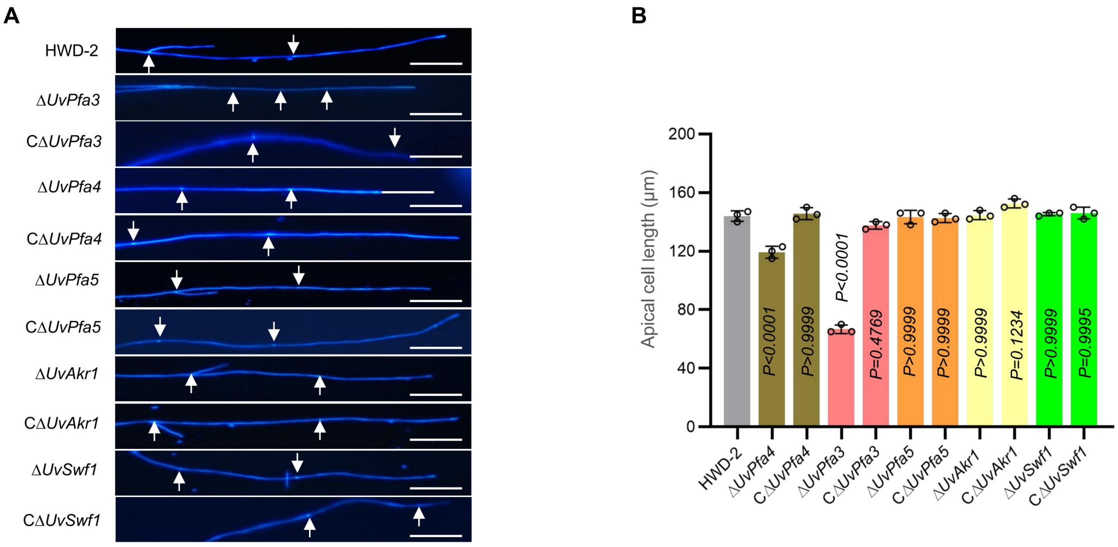

FIG S5 Apical cell lengths of *U.virens* palmityltransferase mutants. (A) Hyphal tips stained by CFW. Scale bar, 60 μm. (B) Measurement of hyphal tip cell lengths of mutant strain.

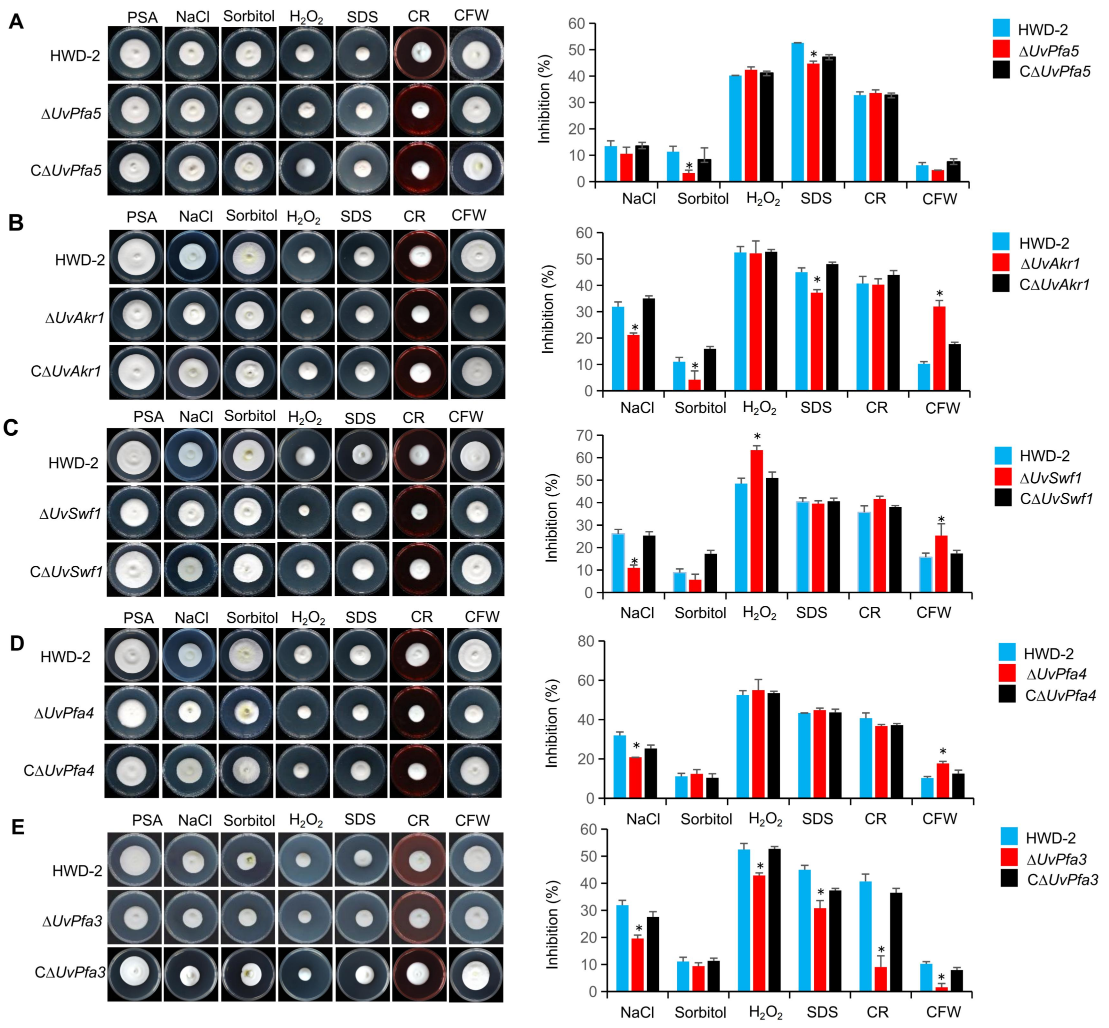

FIG S6 Colony morphology and inhibition of palmityltransferase (A-E) UvPfa5, UvPfa3, UvPfa4, UvSwf1 and UvAkr1 knockout mutants under different stresses. Asterisks indicate statistically significant differences compared to the wild-type ($P < 0.05$).

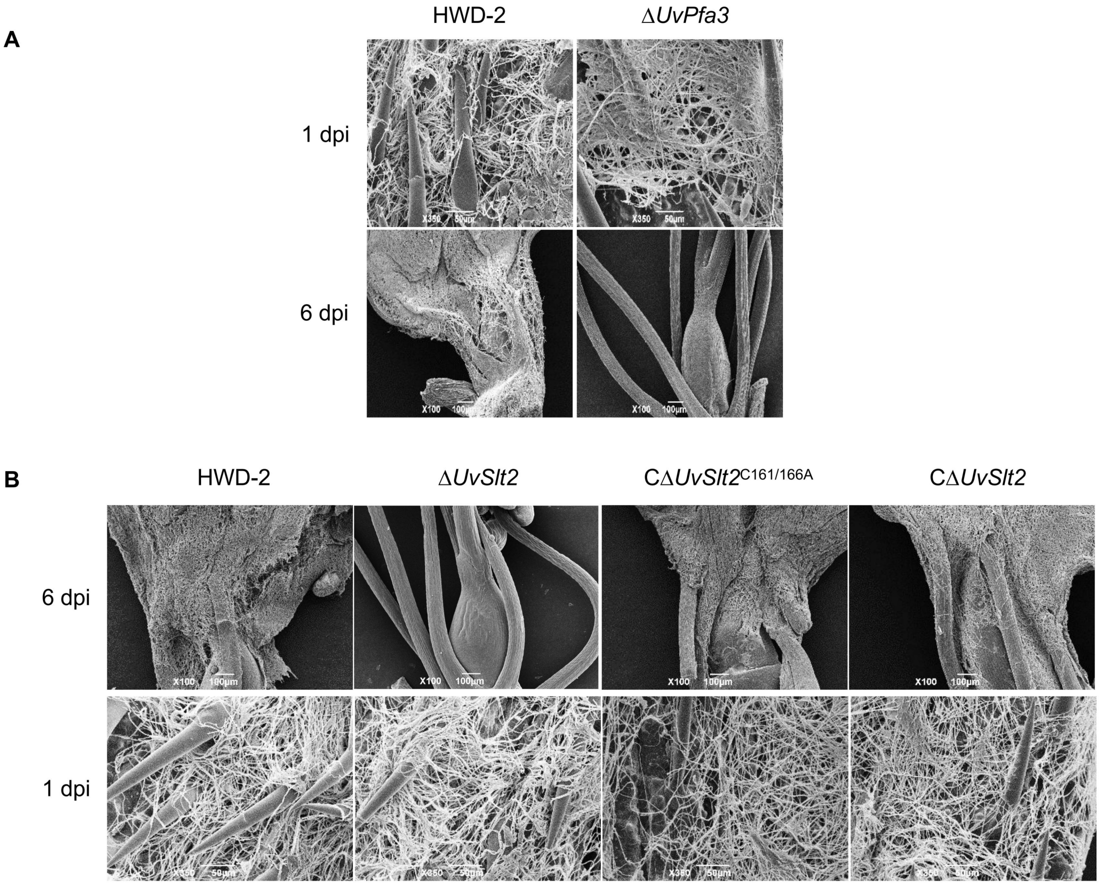

FIG S7 Scanning electron microscopy observation of infected filaments. (A) SEM rice spikelets infected by the palmitoyltransferase UvPfa3 mutant at 1 dpi and 6 dpi. (B) SEM rice spikelets infected by the HWD-2 strain, Δ*UvSlt2* mutant strain, Δ*UvSlt2*$^{C161A/C166A}$ mutant strain and CΔ*UvSlt2* complementary strain at 1 dpi and 6 dpi.

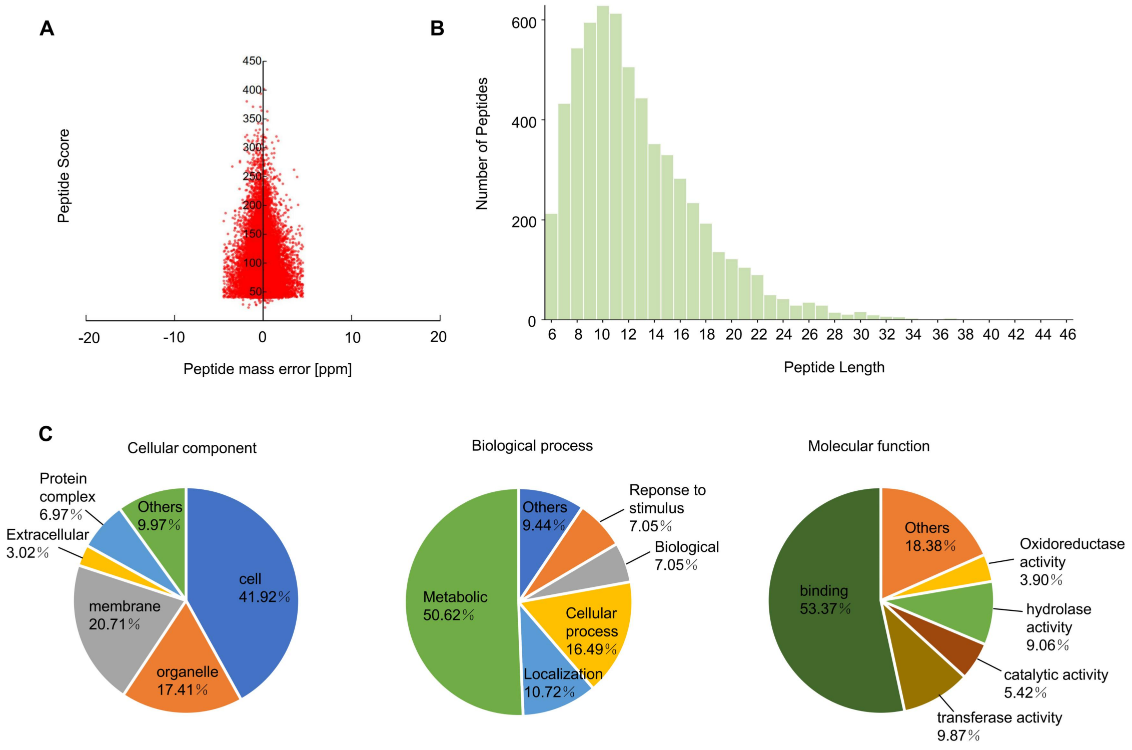

FIG S8 Proteomic analysis of S-palmitoylation in *U. virens*. (A) Length distribution of peptide segments by Mass Spectrometry. (B) Quality accuracy of the mass spectrum data. (C) Analysis of biological processes, molecular functions, and cellular components of S-palmitoylated proteins.

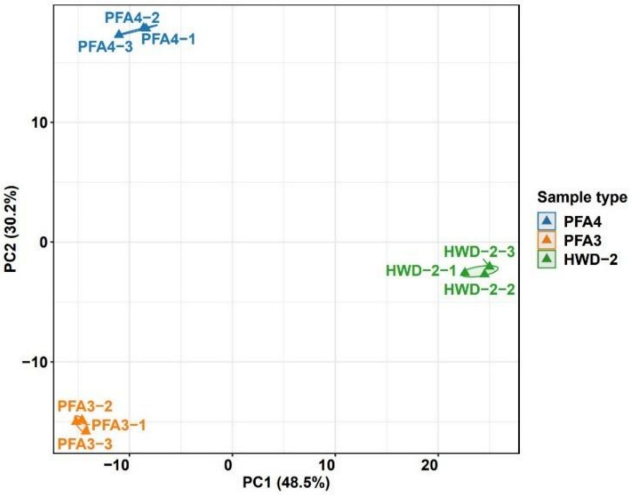

FIG S9 PCA analysis of quantitative proteomics of S-palmitoyltransferases Δ*UvPfa3* and Δ*UvPfa4* mutants.

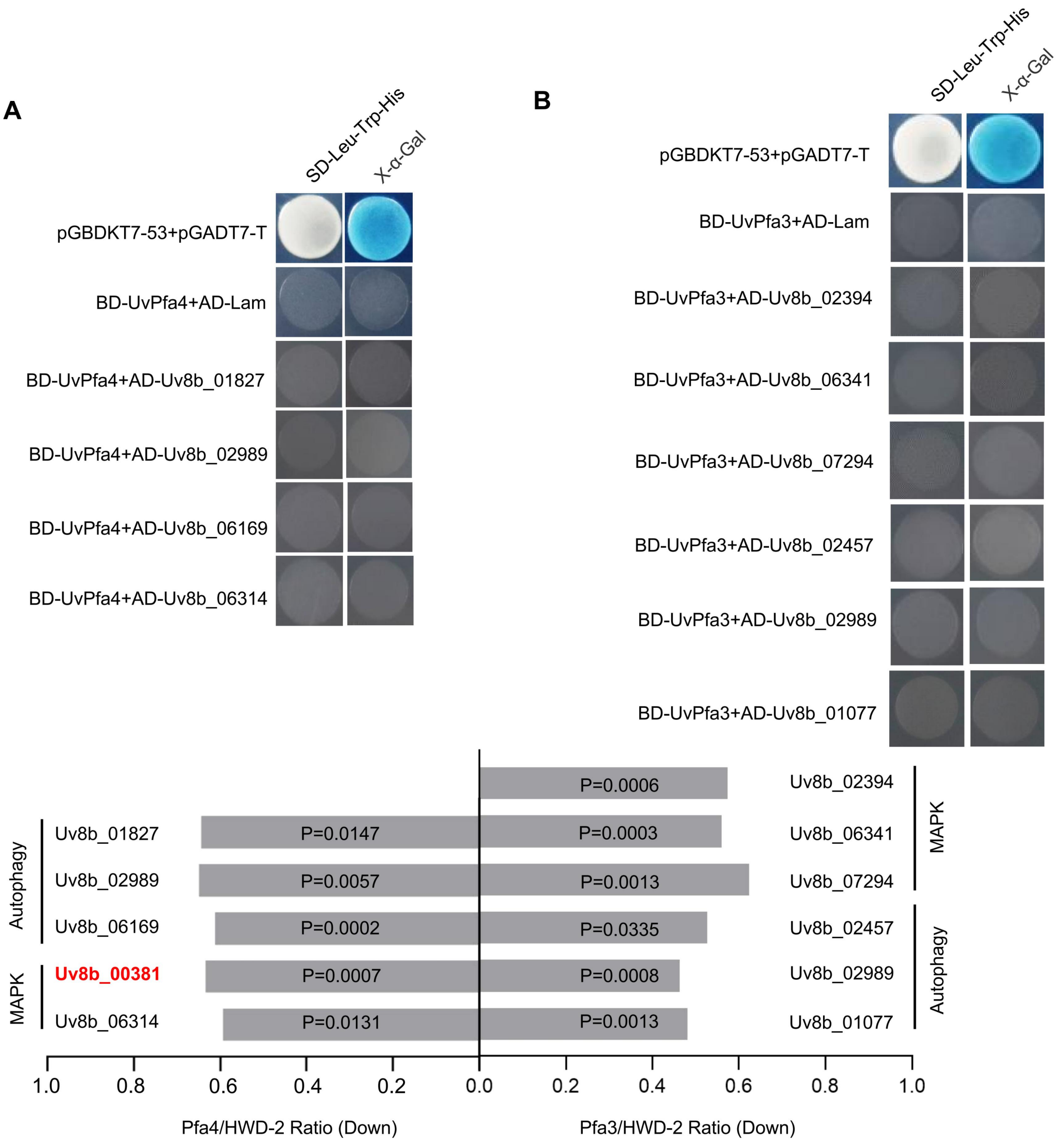

FIG S10 Y2H verification of the interactions between the palmitoyltransferase UvPfa3 or UvPfa4 and selected down-regulated proteins in MAPK and autophagy pathways. (A) Y2H analysis of the interactions between UvPfa4 and its substrates (Uv8b_01827, Uv8b_02989, Uv8b_06169 and Uv8b_06314). pGBKT7-53+pGAD7-T was the positive control; BD-UvPfa4+AD was the negative control. BD, pGBKT7; AD, pGADT7. (B) Y2H analysis of the interactions between UvPfa3 and its substrates (Uv8b_02394, Uv8b_06341, Uv8b_07294, Uv8b_02457, Uv8b_02989 and Uv8b_01077). pGBKT7-53+pGAD7-T was the positive control; BD-UvPfa3+AD was the negative control. BD, pGBKT7; AD, pGADT7.

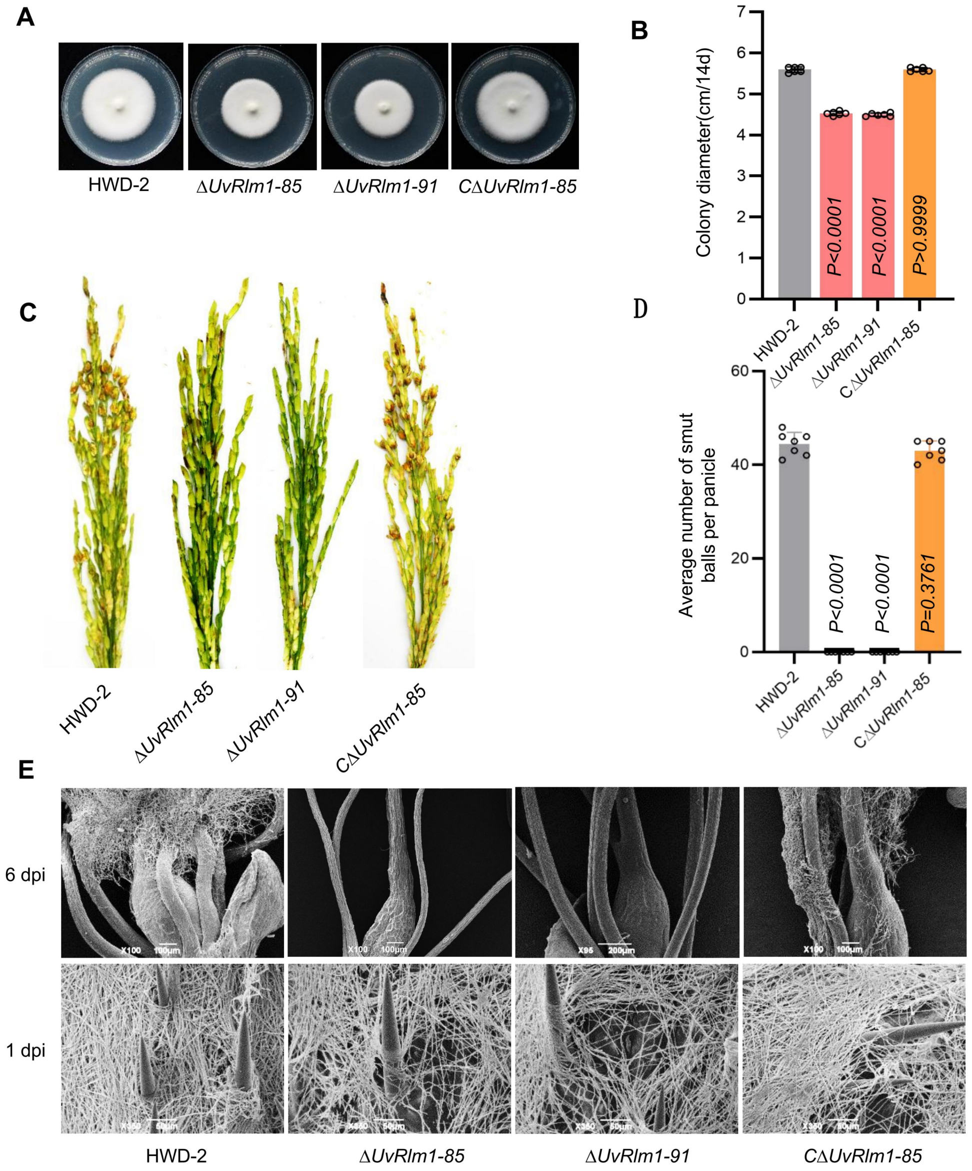

FIG S11 Phenotypic analysis of Δ*UvRlm1* mutant of *U. virens*. (A) Colonies of Δ*UvRlm1* mutants, wild-type strain HWD-2 and CΔ*UvRlm1* complementary strain on PSA for 14 days at 28°C. (B) Colony diameters of Δ*UvRlm1* mutants on PSA after 14 d. (C) Virulence assays of Δ*UvRlm1* mutants on rice spikelets at 21 dpi. (D) Mean number of rice smut balls per panicle. Data were collected from three independent experiments for each treatment. Seven rice panicles were inoculated per replicate. (E) SEM rice spikelets infected by the HWD-2 strain, Δ*UvRlm1* mutants and CΔ*UvRlm1* complementary strain at 1 dpi and 6 dpi.

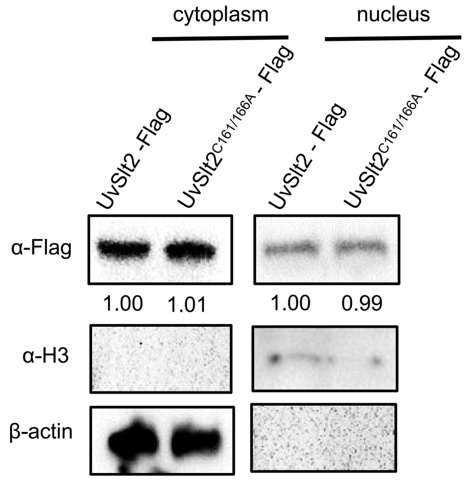

FIG S12 Western blot detection of UvSlt2 and UvSlt2$^{C161A/C166A}$ localization. H3: Nuclear reference; β-actin: Cytoplasmic reference. Anti-Flag antibody was used to display the level of UvSlt2/ UvSlt2$^{C161A/C166A}$ protein and to indicate the relative signal intensity of each band. UvSlt2-flag sample was set to 1.00.
